# Interplay between host and environmental filters drives plant-associated microbiomes in the remote sub-Antarctic Kerguelen Islands

**DOI:** 10.1186/s40793-025-00814-2

**Published:** 2025-12-22

**Authors:** Constance Bertrand, Roland Marmeisse, Marie-Claire Martin, Françoise Binet

**Affiliations:** 1 Univ Rennes, CNRS, ECOBIO [(Ecosystèmes, Biodiversité, Évolution)] - UMR 6553, 35000 Rennes, France; 2 Institut de Systématique, Evolution, Biodiversité (ISYEB), Muséum National d’Histoire Naturelle, CNRS, Sorbonne Université, EPHE, Université Des Antilles, 57 Rue Cuvier, CP39, 75005 Paris, France

**Keywords:** Plant holobiont, Bacteria, Fungi, Sub-Antarctic, Environmental gradient

## Abstract

**Background:**

Plants evolve as holobionts, ecological and evolutionary units made up of the host plant and its associated microbiota, which shape plant fitness and adaptive capacity. Isolated ecosystems with low biodiversity and plant cover, such as the fellfields of the remote sub-Antarctic Kerguelen Islands, represent ideal open-air laboratories to disentangle the drivers affecting plant-microbiome interactions. In such pristine environments, endemic plant species and their microbiota have coevolved in isolation possibly since the last ice age. In this study, we investigated the bacterial and fungal communities associated with different soil–plant compartments of two phylogenetically distant endemic plants, the Poaceae *Poa kerguelensis* and the Brassicaceae *Pringlea antiscorbutica*, in fellfields with contrasted pedoclimatic conditions.

**Results:**

Using 16S rRNA gene and Internal Transcribed Spacer (ITS) region metabarcoding, we identified a strong soil–plant compartment effect affecting microbial communities, with bacterial and fungal α-diversity higher in bulk and rhizospheric soils and progressively decreasing in roots and above-ground compartments. The microbiota of the different soil–plant compartments studied differ in their recruitment patterns. The bacterial communities of the aerial parts of *P. antiscorbutica* were less dependent on those of the underground parts compared to those of *P. kerguelensis*. We also showed that the microbiota of distinct plant species and their different soil–plant compartments respond differently to pedoclimatic variables, with a greater impact of climatic variables over soil ones on aboveground bacterial microbiomes than on belowground microbiomes.

**Conclusions:**

Our results highlight the dual role of environmental variability and of the identity of the host on the recruitment and diversity of plant microbiomes in the isolated studied ecosystems. As plant holobionts are part of the global biogeochemical ecosystem functioning, our results suggest that plant species-specific microbial recruitment strategies and differential vulnerability to environmental factors should be included in predicting sub-Antarctic ecosystem response to global warming.

**Graphical abstract:**

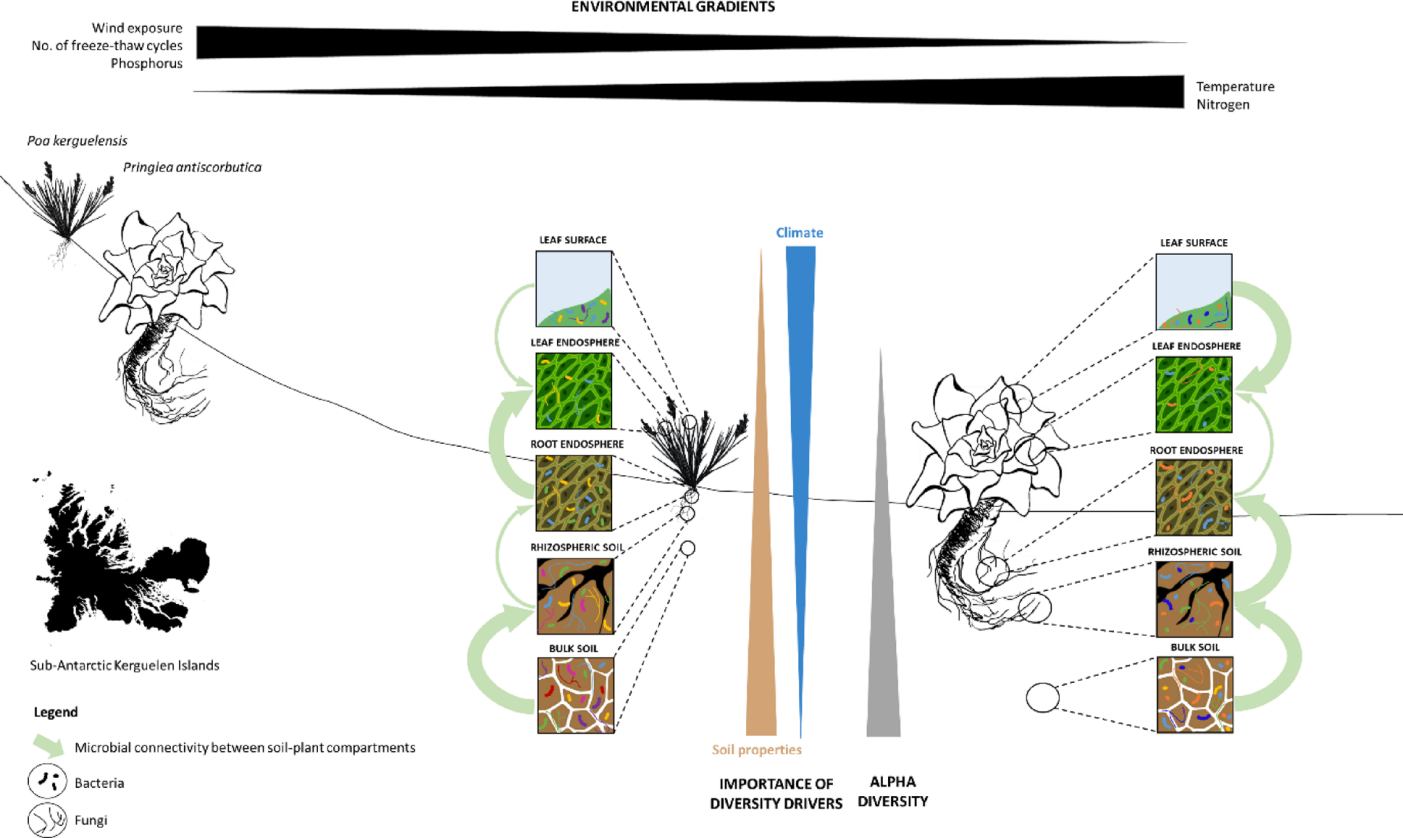

**Supplementary Information:**

The online version contains supplementary material available at 10.1186/s40793-025-00814-2.

## Background

Plants evolve in environments rich in microorganisms that can colonize their rhizosphere (soil under the influence of the root) and all of their organs, including roots, leaves, or seeds [1]. These associated microorganisms are transmitted either vertically from generation to generation [2] or recruited horizontally from the pool of microorganisms in the local environment (*e.g*. air, soil) [3]. The microbiota that interacts with a plant contributes to functions that are crucial for the fitness of the plant; nutrient uptake (notably phosphorus or nitrogen through symbiotic associations), resistance to pathogens, or to abiotic stress [4–8]. These biotic interactions between macro- and microorganisms have led to the emergence of the “holobiont” concept, where the host plant and its associated microbiome constitute a single ecological and evolutionary unit [9].

Plant adaptation to environmental changes thus relies on diverse biological and ecological mechanisms, genetic diversity being the *sine qua non* condition for natural selection and therefore long-term adaptation, while phenotypic plasticity allows individuals to modulate their morphology and physiology in response to environmental fluctuations. As for the plants’ microbiome, it extends the host plant’s functional phenotype by affecting nutrient cycling, stress tolerance, and disease resistance [10–12]. Additionally, microbial communities can display their own ecological dynamics along environmental gradients, further modulating host response to environmental variability [13]. Within this holobiont framework, microorganisms are active and co-evolving partners that influence plant fitness and adaptation. This aligns with the “hologenome theory of evolution”, which postulates that selection acts not only on the plant genome but on the combined genomes of the plant and its associated microorganisms [14].

The current study documents the holobiont concept by investigating the structure and environmental responsiveness of microbes associated with two phylogenetically distant and perennial endemic plant species that co-occur in the same sub-Antarctic habitat currently undergoing rapid climate change.

**Fig. 1:**
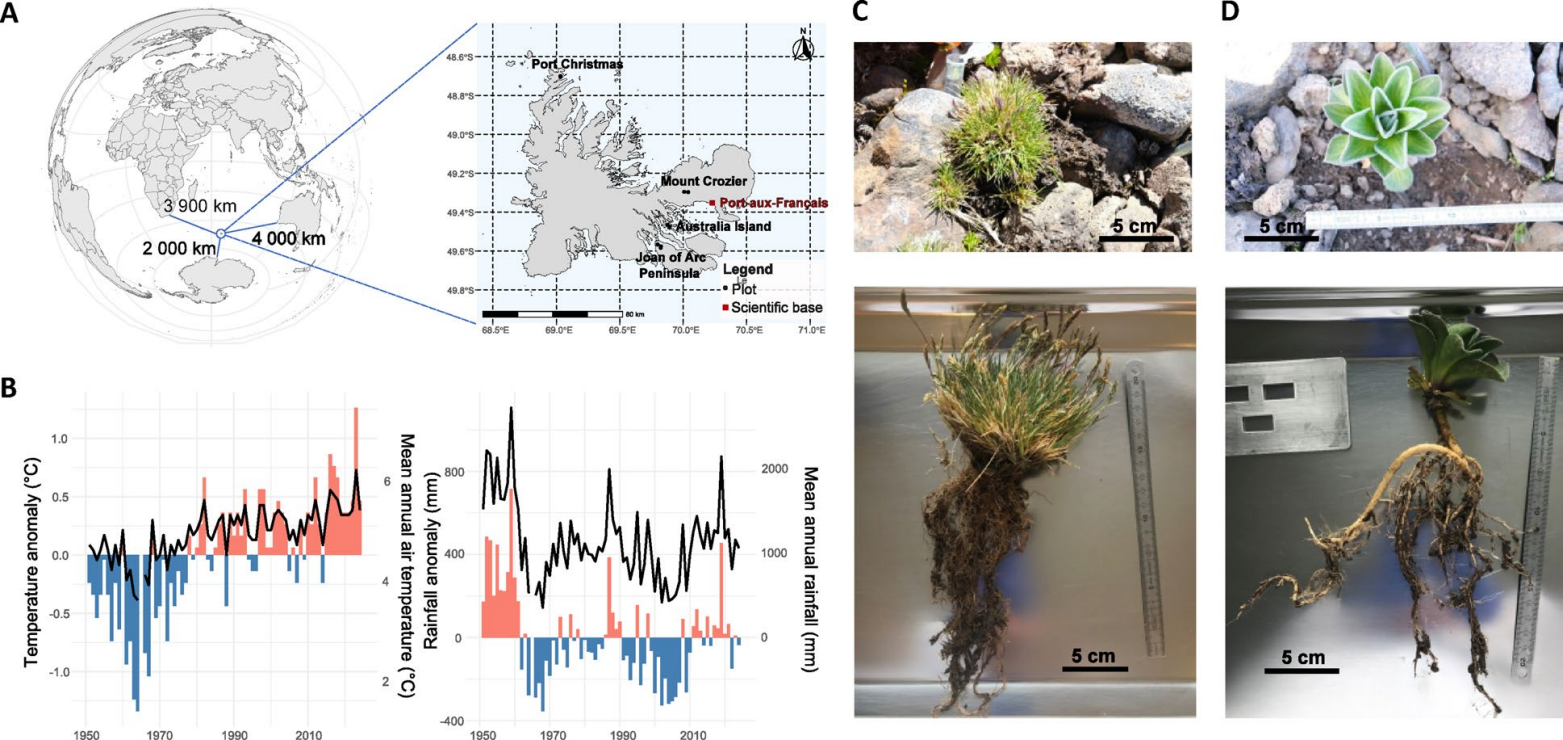
Study sites and studied plant species. Geographic location of the Kerguelen Islands and position of sampling sites (A). Climate change in the Kerguelen archipelago since 1951, as visualized by the evolution of mean annual air temperature and annual rainfall values expressed as deviations from the mean of the 1951–2024 period (Port-aux-Français records by Metéo-France) (B). Whole *Poa kerguelensis* (C) and *Pringlea antiscorbutica* (D) plants

The sub-Antarctic Kerguelen archipelago, one of the most isolated landmasses on Earth (Fig. 1A) and home to very species-poor terrestrial communities [15], is an ideal open-air laboratory to study these plant-microbiota interactions. While diversity of both animal and plants is well documented in this archipelago [16], microbial diversity remains largely unexplored, with only a few sporadic studies targeting the soil microbiota [17–20]. In these islands, fellfields, rocky and windswept areas shaped by glacial retreat [21], represent one of the ecosystems least affected by anthropic activities and biological invasions, with few native plant species (13 among the 22 species native to Kerguelen) as the main or exclusive components of local plant communities. Fellfields thus represent an ideal pristine biotope in which to investigate the impact of environmental variables on local biodiversity.

In fellfield environments, the presumably pre-glacial origin of the local flora [22], the long-term climate stability and isolation may have favored the establishment of tightly integrated plant–microbe associations. These potentially long-lasting ecological assemblages are now facing increasingly rapid environmental changes. Fellfields are indeed experiencing both biotic stresses, such as the multiple introductions of animal and plant species, and abiotic ones, notably climatic shifts linked to global warming [23], rapid and obvious in this region [24, 25]. Since 1951, there has been a sharp increase in mean annual temperatures and a decrease in annual rainfall (+ 0.73°C; −30 mm per decade) (Fig. 1B). These changes have already affected the local fauna and flora, and notably caused community shifts to the benefit of alien species [15, 26, 27]. While the effects of climate change on macroscopic biodiversity are well documented, their impacts on plant and soil microbiomes remain uncertain, despite microorganisms being key regulators of global biogeochemical cycles and climate.

To capture the influence of environmental variation on microbial communities at the local scale, we selected sampling sites along an altitudinal gradient spanning a range of microclimatic conditions. The measured environmental parameters at each site (e.g., soil temperature, soil moisture, soil properties) allowed us to explicitly test the effects of local scale variations in abiotic variables on soil and plant-associated microbiota. The environmental conditions prevailing at the most extreme sites could be representative of those predicted by future climate scenarios, thus serving as proxies for exploring the potential responses of microorganisms to environmental changes.

By characterizing the diversity and structure of fungal and bacterial communities associated with soils and different plant organs in contrasted fellfield ecosystems of the Kerguelen Islands, we hypothesize that, given the sharp decrease in temperature with elevation [28], the composition and diversity of microbial communities should respond significantly to variations in abiotic conditions along the gradients. We also hypothesized that, due to Kerguelen’s isolation and the appearance of native plant species before the glacial maximum, coevolution between host plants and their microbiota has taken place, leading to plant species-specific effects on microbial communities. We further hypothesize that phylogenetically different plant species recruit distinct microbiomes, reflecting different life strategies.

Our field-based study reveals a set of climate and edaphic filters shaping the soil and plant microbiota, with plant species-specific recruitment patterns and grounds perspectives of the plant holobionts coping with climate change.

## Methods

### Plant species

*Poa kerguelensis* Steud. (Poaceae; Fig. 1C), a hardy tussock grass, and *Pringlea antiscorbutica* R. Br. (Brassicaceae; Fig. 1D), commonly known as Kerguelen cabbage, are two perennial species, both endemic to the Southern Indian Ocean Province, that encompasses the Kerguelen, Marion, Prince Edward, Crozet and Heard Islands. Their populations have faced significant anthropic disturbances in the past century. The introduction of rabbits to the Kerguelen Islands led to severe grazing pressure, drastically reducing their presence in many areas. Alien grasses, have further contributed to habitat modification and competition for space and nutrients [29, 30]. Thus, *P. kerguelensis* and *P. antiscorbutica* are now primarily confined to inland fellfields or steep, inaccessible coastal cliffs where they are less vulnerable to grazing and competition with introduced species. Inland fellfields, with strong winds and frequent frost events, represent potential refuge areas where biotic pressures are lower. The two species are now emblematic of these fellfield ecosystems.

### Study sites and plant sampling design

Plants and associated soil were sampled during the 2022–2023 and 2023–2024 austral summers (December to February). Four fellfield sites in the Kerguelen Islands were visited, Mount Crozier (CRO) and Joan of Arc Peninsula (PJA) in the east of the mainland, Australia Island (AUS) in the Morbihan Gulf, and Port-Christmas (PCH) in northwestern Kerguelen (Fig. 1A).

At each site, we selected two *ca*. 10×10 m plots per plant species that differed in elevation to encompass contrasting microclimatic conditions (Table S1). While several plots encompassed both plant species, others did not, resulting in a total of 2 or 3 plots per site. Within each plot, five isolated individuals per plant species were sampled. The low vegetation cover in fellfields allowed for the sampling of isolated plants in order to minimize potential interferences from neighboring vegetation. While *P. kerguelensis* was present and sampled on all four sites, *P. antiscorbutica* populations were only found in CRO and PJA (*i.e.* 8 against 4 populations (plots)).

### Plant traits and plant-soil microbiome sampling

Plants were first measured for several phenotypic and reproductive traits as summarized in Table S2. Each collected plant was then subsampled to analyze separately its rhizospheric soil, root endosphere, leaf endosphere and leaf surface microbiomes (hereinafter referred to as “the soil–plant compartments”). Prior to uprooting the plant, microorganisms on the leaf surface were collected by cleaning with a sterile cotton swab the adaxial side of three leaves per plant. For *P. antiscorbutica*, the three leaves were selected, one in the center, one in the middle and one on the outside of the rosette (Fig. 1D), to account for age-related variations in microbial communities. To minimize contaminations from soil particles, for both plant species, the outermost leaves of the plants were excluded. The swab was then stored in RNAlater™ solution. Five bulk soil cores (0–10 cm depth) dug with a sterilized gouge auger in the vicinity of each sampled plant were pooled as one composite bulk soil sample. Rhizospheric soil was collected by recovering soil aggregates that remained attached to roots after manually shaking the excavated root system. Roots were further cleaned in two successive bathes of sterile water and one of 70% ethanol before being stored in 70% ethanol. Cleaned leaves were directly stored in sterile tubes. All soil and plant samples were frozen at −20°C right after conditioning and stored frozen until DNA extractions.

### In situ plot instrumentation and abiotic soil-climate measurements

Air (2 cm and 15 cm above soil surface) and top soil (at a 6 cm depth) temperatures and relative soil moisture were recorded every 15 min in each plot with a TMS4 datalogger (TOMST, Czech Republic; [31]) deployed on each plot since December 2022. Mean soil temperature, soil moisture, and the number of freeze–thaw cycles were calculated for the period during which the majority of all TMS4 probes were simultaneously operational, from January 27, 2023, to January 16, 2024. Average and maximal wind speed over a one-hour timespan at every plot were measured with a portable anemometer (Kestrel 1000 wind meter, Kestrel Instruments®, Boothwyn, PA). Each measure was standardized with respect to the hourly average wind speed measured at the weather station of the scientific base Port-aux-Français for the same timespan to obtain a comparable wind exposure index for each plot.

Bulk soil samples were analyzed for total nitrogen, total carbon, C/N ratio, available Olsen phosphorus, soluble sulfur, nitrates, ammonium and pH following standardized protocols (INRAE Soil Analyses Laboratory, Arras, France; https://las.hautsdefrance.hub.inrae.fr/). Two bulk soil samples per site were also analyzed for texture, total and exchangeable micronutrients contents (Fig.S1, Table S1).

### DNA extraction and metabarcoding

DNA extractions were performed using Qiagen extraction kits (Power Soil Pro Kit and Plant Pro Kit for soil and plant samples, respectively) according to the manufacturer’s instructions. Roots and leaves were first surface-sterilized by successive passages through different baths of 70% ethanol and diluted bleach according to Wemheuer and Wemheuer [32] and ground in liquid nitrogen with a mortar and pestle prior to the extractions. Absence of external contaminants was assessed by PCR amplification using aliquots of the last surface sterilization bath water as template. A pre-extraction step was necessary for the swabs, for which we followed the protocol by Walker et al. [33]. DNA concentration and quality were assessed using a SpectroSTAR spectrophotometer (BMG Labtech, France).

Metabarcoding of bacterial and fungal communities was performed by amplifying and sequencing the V4 region of the 16S rRNA gene (primers 16S 515F 5’-GTGYCAGCMGCCGCGGTAA-3’ and 16S 806R 5’-GACTACHVGGGTWTCTAAT-3’; [34–36]), and the fungal ITS2 region of the Internal Transcribed Spacer (ITS) (primers FITS9 5’-GAACGCAGCRAAIIGYGA-3’ and ITS4 5’-TCCTCCGCTTATTGATATGC-3’; [37]), respectively. Due to the low amounts of DNA extracted from leaves and leaf surfaces, we did not amplify and sequence fungal ITS2 regions from these samples. All locus-specific primers were extended by Illumina tails and PCR amplifications, indexing and sequencing were performed by IGA Technology (Udine, Italy) on a NovaSeq6000 instrument (Illumina, San Diego, CA) using 2 × 250-bp paired-end mode. In the case of root and leaf DNA extracts, Peptide Nucleic Acid (PNA) clamps were added to the PCR amplification mix to limit amplification of plant chloroplast and mitochondrial 16S rRNA gene sequences (PNA Bio Inc, Newbury Park, CA).

### Raw sequence preprocessing

All sequence preprocessing steps and statistical analyses were carried out with R (v. 4.3.0, R Core Development Team, 2005; www.R-project.org).

Raw sequences were demultiplexed and primer-trimmed with cutadapt [38]. Reads were then processed using DADA2 [39]. Because of the large number of amplified sequence variants (ASVs), sequence alignments were performed with MAFFT (v. 7.520; [40]) and phylogenetic trees were computed with FastTree2 (v. 2.1.11; [41]). While bacterial sequences were kept as ASVs, fungal ITS sequences were clustered as OTUs at a 97% identity threshold with the DECIPHER package [42], in order to take into consideration intraspecific polymorphisms. ASVs and OTUs taxonomic affiliation was performed using SILVA (v.138.1; [43]; https://www.arb-silva.de/) and UNITE (v.10.0; [44]; https://unite.ut.ee/) databases, respectively.

From the total datasets, containing 147,876 bacterial ASVs and 19,818 fungal OTUs, we filtered out 16S rRNA sequences affiliated to plant chloroplasts or mitochondria, as well as ITS sequences affiliated to non-fungal taxa as well as ITS and 16S rRNA gene sequences that could not be affiliated to any Kingdom or Phylum. We also eliminated from the analysis ASVs and OTUs represented by sequences whose abundance represented less than 0.005% of the total dataset. Additionally, we removed putative contaminants using with the microDecon package [45] that makes use of sequences amplified from the extraction blank samples. The final dataset included 3,682 fungal OTUs and 61,340 bacterial ASVs.

Sequencing depth was assessed for each sample to ensure sufficient coverage of microbial diversity. Rarefaction curves were generated for each of samples using the vegan R package, showing that all sample datasets reached a plateau (Fig. S2).

### Statistical analyses

To account for the compositional nature of amplicon sequencing data without discarding information through rarefaction, we applied a centered log-ratio transformation and computed Euclidean distances (thereafter referred to as Aitchison distances) for β-diversity analyses [46, 47]. For α-diversity analyses, we used the Shannon index, as it is relatively robust to differences in sequencing depth, with non-significant correlations between Shannon index and sequencing depth (Pearson; p > 0.05), and accounts for both species richness and evenness.

Effects of plant species, sampled plot and soil–plant compartment were evaluated on Shannon indices with Kruskal–Wallis tests followed by a Dunn test for multiple comparisons; and on Aitchison distances with 9999 permutations PERMANOVAs using the vegan package.

The nestedness of microbial communities across sampled compartments of all sites combined was assessed using the vegan package. This analysis aimed to quantify the degree of taxonomic overlap between compartments and to visualize taxa that are shared between soil–plant compartments versus taxa that are compartment-specific. This analysis, based on the presence-absence of taxa, produces a temperature metric (T) that indicates the degree of nestedness between soil–plant compartments. T ranges from 0 to 100; zero indicates a perfect nestedness, where taxa are hierarchically shared across compartments while 100 indicates a random distribution of the taxa in the different compartments. This analysis also returns a matrix fill value representing the proportion of ASVs or OTUs present across all soil–plant compartments, reflecting overall taxonomic occupancy.

Source tracking analyses, based on the abundance of each taxa (bacterial ASVs or fungal OTUs) in each soil–plant compartment, were conducted using SourceTracker2 [48] to estimate the origin and potential transmission of bacterial and fungal molecular taxa between adjacent soil–plant compartments. A sink-source model was used where each compartment was alternatively considered as a sink (recipient community) and the adjacent ones as potential sources.

The influence of biotic (plant morphology) and abiotic (climate and soil parameters) factors on microbial α-diversity (Shannon index) across soil–plant compartments was assessed using linear mixed-effect models taking the plot as a random effect. The best-fitted model was selected via stepwise backward model selection based on the Akaike Information Criterion (AIC). Multicollinearity among predictors was evaluated using Variance Inflation Factor (VIF < 5), and assumptions of normality and homogeneity of variance were tested using the Shapiro–Wilk and Breusch-Pagan tests, respectively.

Effects of climatic, edaphic and plant morphology variables on microbial community assembly were tested using 9999-permutations PERMANOVAs on Aitchison distances. The relationship between Aitchison and geographic distances was modeled using a negative exponential function.

In order to rank by importance the effect of these variables on both α and β diversity, we established structural equation models (SEMs) using the package piecewiseSEM [49] across all sampled soil–plant compartments. SEMs were constructed with linear mixed-effects models, incorporating the plot as a random effect to account for spatial non independence of samples collected in the same plot. For each model within the SEM, we assessed normality and homogeneity of variance using the Shapiro–Wilk and Breusch-Pagan tests, respectively. We also assessed multicollinearity among predictors using variance inflation factors (VIF). Climatic and edaphic variables were selected based on their significance in prior alpha and beta diversity analyses, and summarized using the values of the first principal component axis of each corresponding PCA, which explained 63.5% and 36.4% of the variance, respectively. To account for host morphological variability, we derived a plant morphology index from the PC1 of a PCA integrating Z-score standardized height, largest diameter, and number of leaves for each plant species, explaining 58.2% of variance. For each soil–plant compartment’s β-diversity, we used the coordinates of the first axis of the PCoA on Aitchison distance. For each soil–plant compartment α-diversity, the Shannon index was used. The models’ goodness of fit was ensured according to the criterion of a low χ2, a non-significant probability level (p > 0.05), and on the base of the lowest AIC.

## Results

### Plot, plant species and soil–plant compartment effects on microbial diversity

**Fig. 2:**
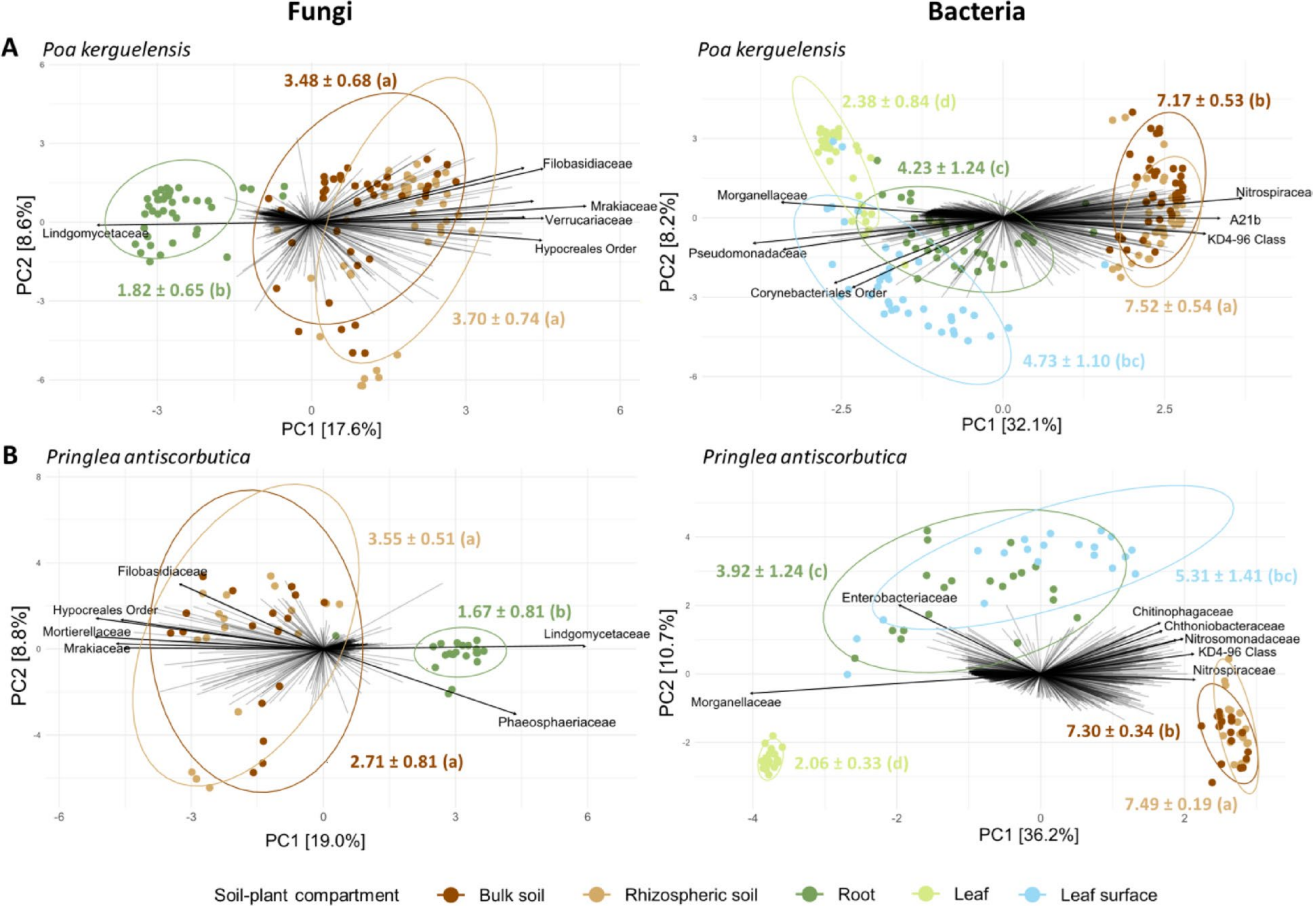
Principal Coordinates Analysis (PCoA) ordinations of fungal (left panels) and bacterial (right panels) communities associated with the different soil–plant compartments of *Poa kerguelensis* (A) and *Pringlea antiscorbutica* (B). Each point represents a sample, and colors correspond to distinct soil–plant compartments: bulk soil, rhizospheric soil, root endosphere, leaf surface, and leaf endosphere. The Aitchison distance was used as the metric of β-diversity. Colored numbers correspond to the average Shannon index values ± standard deviation for each compartment and different letters indicate significant differences between compartments (p < 0.05). Arrows points to the bacterial and fungal families or orders contributing most to communties’ dissimilarity. In the case of bacterial communities, the third axis of the PCoA clearly separated the root from the leaf surface communities (Fig. S3)

Although the two plant species studied are phylogenetically distant, we did not observe any significant effect of plant species on α-diversity (using Shannon’s index as a metric) of associated bacterial and fungal communities (Kruskal–Wallis; p > 0.05). The same observation was made for the plots, which had no significant effect on α-diversity (Kruskal–Wallis; p = 0.05). However, a strong effect of the soil–plant compartment was observed (bacteria; χ^2^ = 241.72, df = 4, p < 0.001; fungi, χ^2^ = 93.50, df = 2, p < 0.001), indicating that each of the soil–plant compartments represented a distinct habitat for the microbial communities. For both bacteria and fungi, soils (bulk and rhizospheric) were characterized by a higher α-diversity, with Shannon index values ranging from approximately 7 to 7.5 for bacteria, and around 3 for fungi. In comparison, roots had lower Shannon indices (bacteria: *ca.* 4; fungi: < 2). Regarding leaves, the bacterial diversity of the leaf surface was similar to that of the roots, while the leaf endosphere consistently displayed the lowest diversity levels, with Shannon indices of about 2 (Fig. 2).

Unlike α-diversity, bacterial and fungal β-diversity (Aitchison distances) was affected not only by the soil–plant compartments, but also by the plant species, the plots and their interactions (Table 1). Among those factors, the plots and soil–plant compartments had the strongest effects on β-diversity, both when all samples were analyzed together or when analyses were performed separately for each plant species.

**Table 1:** PERMANOVAs revealing effects of soil–plant compartments, plots, plant species and their interactions on microbial communities’ assembly (Aitchison distances)

	Bacteria				Fungi			
	df	F	R^2^	p	df	F	R^2^	p
*Global*								
Plot	8	5.8568	0.09730	0.0001	8	4.4221	0.15117	0.0001
Soil–plant compartment	4	16.6832	0.13857	0.0001	2	6.1014	0.05214	0.0001
Host species	1	2.1248	0.00441	0.0482	1	4.6564	0.01990	0.0004
Plot × Soil–plant compartment	32	3.4291	0.22787	0.0001	16	2.0257	0.13850	0.0001
Plot × host species	2	–	–	–	2	2.1689	0.01854	0.0051
Soil–plant compartment × host species	4	1.7231	0.01431	0.0185	2	–	–	–
Plot × Soil–plant compartment × host species	8	1.34375	0.02388	0.283	4	–	–	–
*Poa kerguelensis*								
Plot	7	5.1697	0.11459	0.0001	7	3.9656	0.17752	0.0001
Soil–plant compartment	4	10.0077	0.12676	0.0001	2	4.2637	0.05453	0.0001
Plot × Soil–plant compartment	28	2.8777	0.25515	0.0001	14	1.7918	0.16042	0.0001
*Pringlea antiscorbutica*								
Plot	3	3.1220	0.05919	0.0002	3	2.4334	0.25442	0.0002
Soil–plant compartment	4	11.1665	0.28225	0.0001	2	4.5594	0.18208	0.0001
Plot × Soil–plant compartment	12	2.3514	0.17831	0.0001	6	1.5824	0.05543	0.00037

Only significant effects (p < 0.05) are reported

All pairwise comparisons between soil–plant compartments highlighted significant differences between the microbial communities (both fungal and bacterial) that colonized them (PERMANOVA; p < 0.01) (Table S3). The only exception was the absence of significant difference between communities in bulk and rhizospheric soils for *P. kerguelensis* (both fungal and bacterial) and *P. antiscorbutica* (only bacterial) (Table S3 and Fig. 2).

**Fig. 3:**
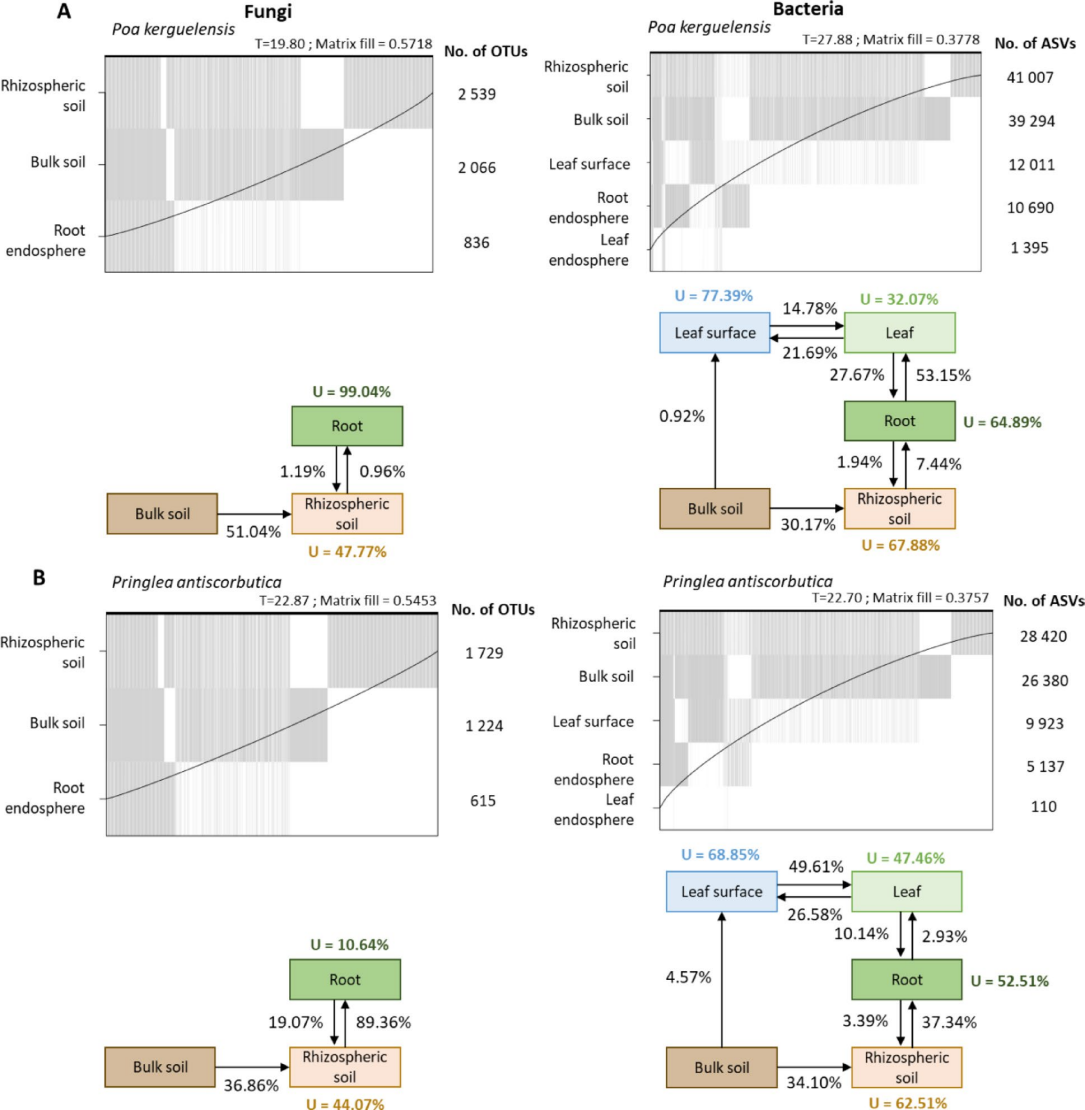
Nestedness and source tracking analyses of fungal (left panels) and bacterial (right panels) communities associated to *Poa kerguelensis* (A) and *Pringlea antiscorbutica* (B). Each grey vertical line in the nestedness plots corresponds to an ASV or OTU that was observed in the corresponding compartment; the curve shows the isocline of perfect nestedness, with grey lines underneath the curve indicating disorder that contributes to the T metric. A lower T value reflects a more nested community structure. The matrix fill parameter reflects the proportion of occupied cells in the matrix. For source tracking analyses, only direct source-sink relationships were tested. The "U" percentage indicates the proportion of reads originating from unknown sources in each soil–plant compartment

Nestedness analysis revealed that microbial communities across soil–plant compartments are not independent of one another. Specifically, root and leaf communities largely represent subsets of the bulk and rhizospheric soil communities (Fig. 3). We then performed source tracking to assess, for each plant species, the contribution of communities in adjacent compartments to communities in compartments connected to them. This analysis revealed profound differences between plant species (Fig. 3). For example, a high proportion of fungal (89%) and bacterial (37%) taxa associated with *P. antiscorbutica* roots appeared to originate from the rhizospheric soil, whereas these proportions are only 1% (fungi) and 7% (bacteria) in the case of *P. kerguelensis* roots (Fig. 3). In the case of the leaf endosphere, while for *P. antiscorbutica* only 3% of bacterial taxa appeared to originate from the roots, this percentage is of 53% for *P. kerguelensis* leaves, which recruit fewer taxa from their surface (15%), unlike cabbage leaves (50%).

These differences in modes of recruitment by the organs of different plants are also reflected in the varying proportions of microbial taxa specific to a given soil–plant compartment (U value in Fig. 3). These taxa are often among the most abundant in the compartment under consideration, and are either completely absent or very poorly represented in the adjacent compartments. Looking at taxa with a minimum of 50% prevalence in root samples and a global relative abundance of at least 0.01% in roots, we noticed that the roots of *P. kerguelensis* are heavily colonized by fungal OTUs that were not detected at all in adjacent soil and by bacterial ASVs that were either not detected or at very low frequencies in soil (Table S4). *P. antiscorbutica* also hosted prominent “root-specific” fungal and bacterial taxa (Table S5). All these “root-specific” fungal OTUs in both plant species belonged to either the Helotiales or Pleosporales, whereas the most abundant root-specific bacterial ASVs had more diverse taxonomic affiliations, predominantly from the orders Pseudomonadales and Burkholderiales.

### Fungal and bacterial α-diversity responds to different environmental cues

**Fig. 4:**
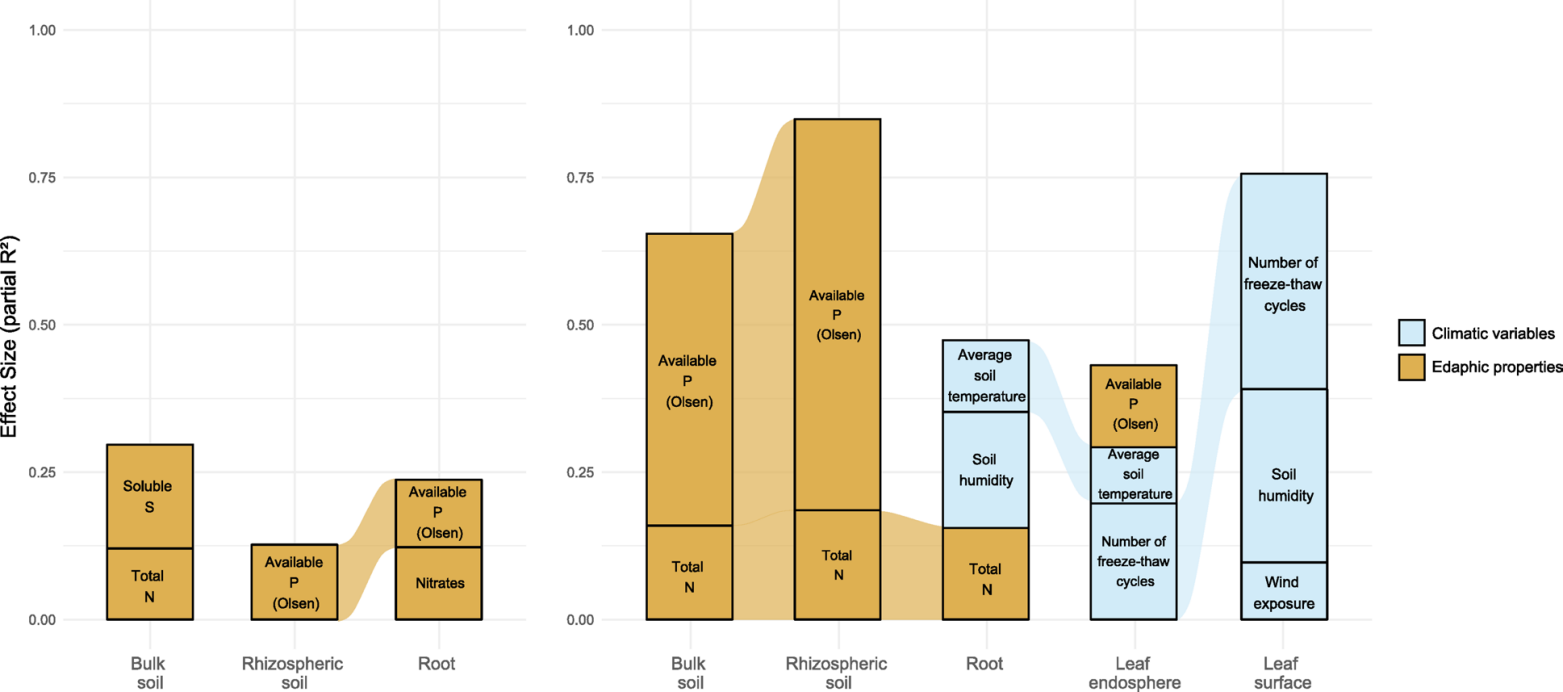
Relative influence of environmental variables on the fungal (left) and bacterial (right) Shannon indices calculated for each of the studied soil–plant compartments. Partial R^2^ values reflect the relative influence of the variables tested individually on the Shannon indices. Only variables with a significant effect (p < 0.05) are reported. Coloured links between bars indicate the effect size of the same explanatory variable across adjacent soil–plant compartments

The sampled plots located in distinct geographic sites of the Kerguelen Islands differed in their climatic and soil characteristics (Fig. S1B; Table S1), making it possible to evaluate the environmental factors that locally affected microbial diversity. Linear models revealed that fungal and bacterial Shannon indices were affected by distinct abiotic variables, with varying contribution of edaphic versus climatic environmental filters in the different soil–plant compartments, but with no influence of plant morphology (Table 2; Fig. 4).

**Table 2:** Linear models assessing the influence of environmental (edaphic and climatic) variables on Shannon indices across soil–plant compartments

Soil–plant compartment	Variables	df	F	R^2^	p	
*Fungi*						
Bulk soil	Total Nᵃ + Soluble Sᵇ	46	5.033	0.1795	0.01056	
Rhizospheric soil	P (Olsen)ᵇ	52	7.574	0.1271	0.00813	
Root	Nitratesᵃ + P (Olsen)ᵃ	51	4.913	0.1615	0.01119	
*Bacteria*						
Bulk soil	Total Nᵃ + P (Olsen)ᵇ	52	26.900	0.4896	9.528e-09	
Rhizospheric soil	Total Nᵃ + P (Olsen)ᵇ	51	51.99	0.6709	4.909e-13	
Root	Total Nᵇ + Average soil temperatureᵃ	47	8.567	0.2672	0.00067	
Leaf endosphere	P (Olsen)ᵇ + Average soil temperatureᵇ + No. of freeze–thaw cyclesᵇ	41	3.753	0.158		0.01802
Leaf surface	Wind exposureᵃ + Soil humidityᵇ + No. of freeze–thaw cyclesᵇ	46	11.020	0.4181	1.425e-05	

Superscript letters indicate the direction of the relationship for each variable with ᵃ indicating a positive effect (estimate > 0, p < 0.05) and ᵇ indicating a negative one (estimate < 0, p < 0.05)

While soil bacterial and fungal communities (bulk and rhizospheric) are mainly affected, either positively or negatively, by soil nutrient availability (P, S, N concentrations), communities associated with plant organs are in addition largely affected by local climatic variables (soil humidity that reflects precipitations, temperature, freeze/thaw cycles frequency). The pre-eminence of climatic over edaphic effects is particularly evident when we consider bacterial communities associated to leaves that are not in direct contact with soil.

In soils, fungal and bacterial communities are not affected to the same degree by the edaphic variables taken into consideration. For example, an increase in soil N concentrations has a positive effect on bacterial bulk and rhizospheric soil α-diversity (β = 0.063, p = 0.0028 and β = 0.055, p < 0.001, respectively), while there was no such effect for fungal diversity. Interestingly, in the root compartment, nitrogen concentration had a negative effect on bacterial diversity (β = -0.164, p = 0.005), while roots’ fungal diversity responded positively to nitrate concentrations in soil (β = -0.087, p = 0.013). On the other hand, the concentration of available phosphorus (Olsen) has a strong negative impact on soil diversity, in bulk soil for bacterial diversity (β = -9.95, p = 0.017) and in rhizospheric soils for both bacteria and fungi (fungi, β = -3.5675, p = 0.008; bacteria, β = -5.93, p < 0.001).

### Environmental and biological filters structure microbial communities’ assembly.

In addition to α-diversity, we also assessed the impact of geographic distance and environmental factors as well as of plant traits on microbial community assembly (β-diversity). Since β-diversity patterns varied not only across soil–plant compartments but also between the two host plant species (Table 1), the effects of spatial distances, environment and plant traits on community structuring were analyzed separately for *P. kerguelensis* and *P. antiscorbutica*.

A significant distance-decay relationship was observed for bacterial and fungal communities in bulk soils and rhizospheric soils for both species, with increasing dissimilarity between distantly located sites (Figs. S4 and S5). No such pattern was observed for root endophytic communities whether bacterial or fungal. For *P. kerguelensis*, endophytic leaf communities differentiated with distance but not those of *P. antiscorbutica* leaves that remained unaffected. Regarding leaf surface communities, both species harbored geographically structured bacterial communities, with *P. antiscorbutica* leaf surface communities displaying the strongest distance-decay relationship (R^2^ = 0.64).

These spatial patterns suggest that microbial assemblages are not randomly distributed but are structured by geographic distance, likely reflecting underlying habitat heterogeneity across plots and geographic sites in the Kerguelen.

**Fig. 5:**
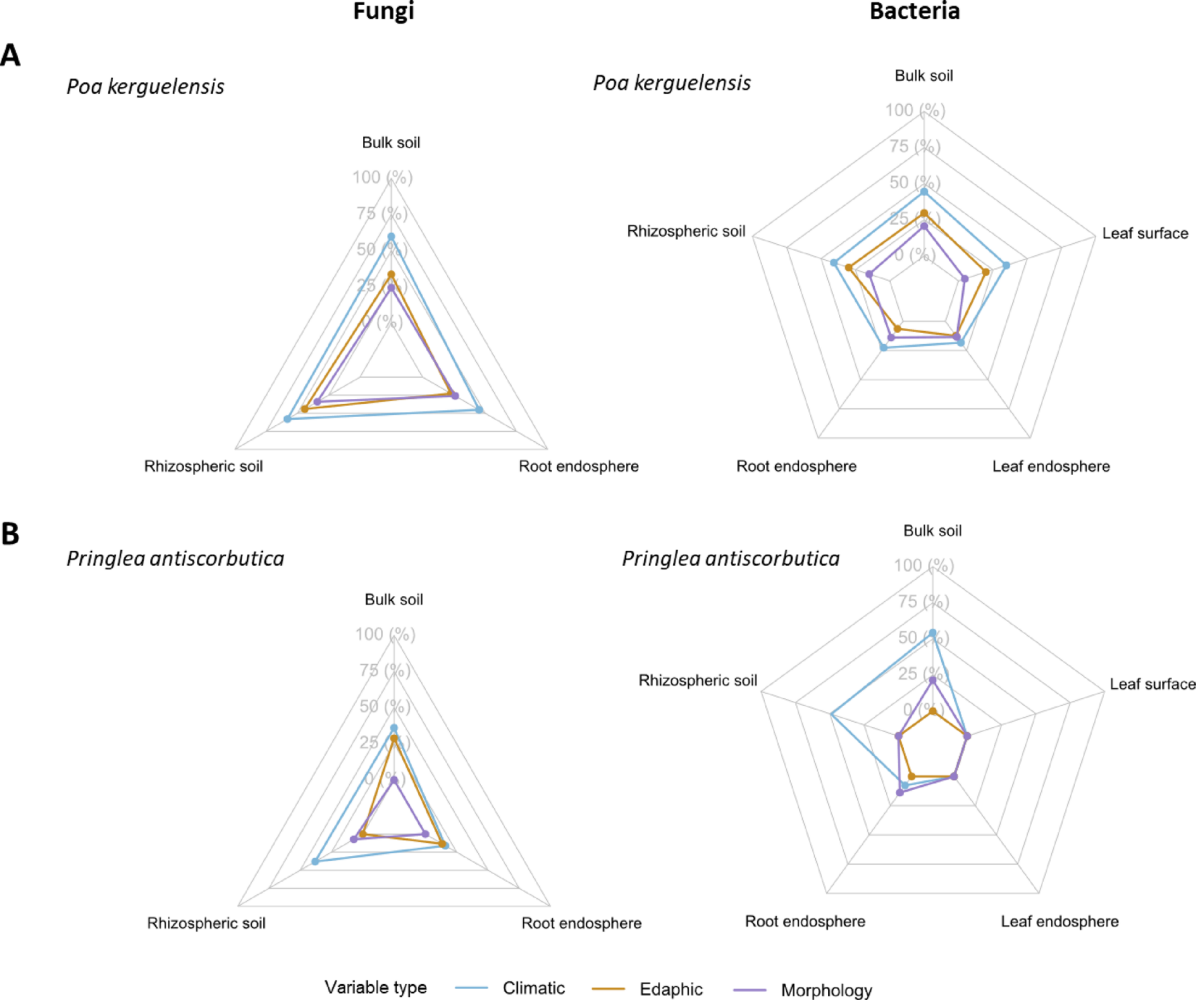
Relative contribution of climatic, edaphic and plant-derived variables to the assembly of fungal and bacterial communities. PERMANOVA’s R^2^ values agglomerated per variable type (*i.e.* climatic, edaphic and morphology) across each sampled compartment, for fungal (left) and bacterial (right) communities for *P. kerguelensis* (A) and *P. antiscorbutica* (B). See detailed PERMANOVAs outputs in Tables S6 and S7

PERMANOVA analyses using Aitchison distances, conducted separately for each plant species and soil–plant compartment, revealed plant species-specific patterns in microbial community assembly, with distinct environmental and morphological drivers affecting diversity across soil–plant compartments (Fig. 5, Tables S6 and S7). Globally, whatever the plant species, microbial communities were primarily structured by climatic variables, followed by edaphic factors, and to a lesser extent by plant morphological traits (Fig. 5).

In *P. kerguelensis*, climatic variables consistently explained the highest proportion of community variation for both fungi and bacteria, especially in bulk and rhizospheric soils. Edaphic variables such as pH, phosphorus, and sulfur also contributed significantly. Plant morphological traits had weaker effects, mainly in the rhizosphere and root compartments.

In contrast, microbial communities in *P. antiscorbutica* showed stronger responses to individual climatic variables, with predictors such as wind exposure and elevation explaining up to 18.6% and 17.4% of the variation in bulk soil bacterial community composition, respectively. Plant morphological variables played a more prominent role in this plant species, unexpectedly in the surrounding bulk soil and in roots (*e.g.,* number of leaves R^2^ = 0.11; diameter at collar R^2^ = 0.10, respectively).

### Ranking the effects of biotic and abiotic variables on α-diversity and microbial community assembly

**Fig. 6:**
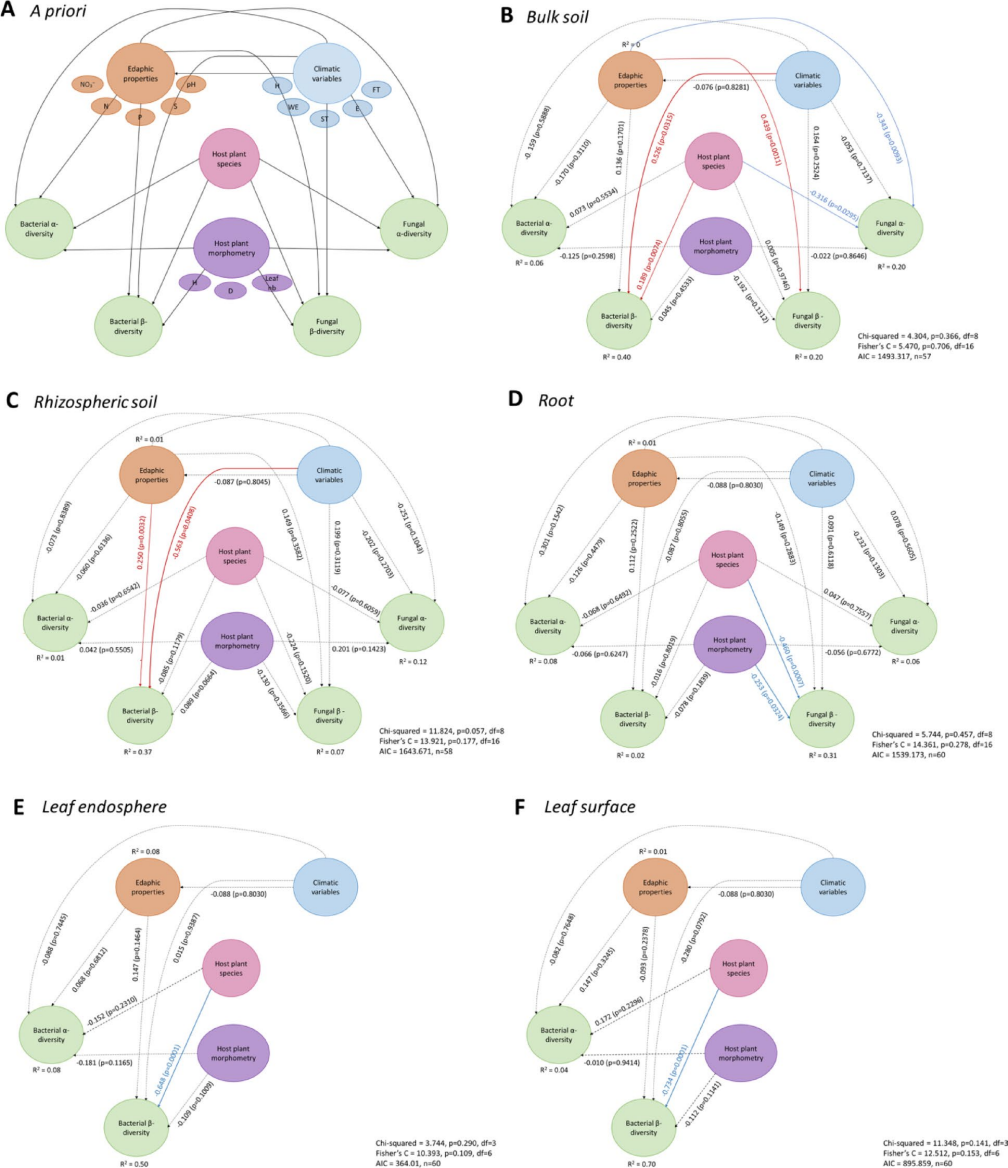
A priori structural equation model (SEM) and outputs for all soil–plant compartments. The climate proxy corresponds to the first PCA axis taking into account soil average temperature, wind exposure, soil humidity, the number of freeze–thaw cycles and elevation (A). The edaphic properties proxy corresponds the coordinates of the first axis of the PCA taking into account soil total nitrogen, nitrates, available phosphorus, soluble sulfur and pH (A). The morphometric proxy corresponds to the first axis of the PCA integrating normalized plant height, largest diameter and the number of leaves (A). Red solid arrows indicate significant positive effects, blue solid arrows indicate significant negative effects, and dashed arrows correspond to non-significant effects (p > 0.05)

Structural Equation Modeling (SEM) conducted separately for the different soil–plant compartments disentangled the relative importance of previously tested parameters on communities’ α-diversity and assembly (Fig. 6). Once again, our findings underline that the influence of environmental and host-related factors on microbial α- and β-diversity is highly compartment-dependent with a shift in the relative importance of drivers from belowground to aboveground compartments.

In bulk and rhizospheric soils (Fig. 6B and C), microbial community assembly was shaped by both climate and edaphic properties, but with compartment-specific patterns. In bulk soil, fungal communities were primarily influenced by edaphic factors (estimate = 0.44, p < 0.01), while bacterial communities responded strongly to climate (estimate = 0.53, p = 0.03) and to soil properties (estimate = 0.53, p = 0.03). In the rhizospheric soil, bacteria were affected by both climatic (estimate = –0.57, p = 0.04) and edaphic variables (estimate = 0.25, p < 0.01), while fungal communities showed no significant response. Host-related variables had minimal effects here, indicating that soil communities are largely structured through abiotic environmental filtering.

In the roots (Fig. 6D), the structuring drivers shifted. The plant species identity and plant traits, possibly reflecting plant’s age, significantly influenced fungal β-diversity (estimate = -0.46, p = 0.007; estimate = -0.25, p < 0.05, respectively), while bacterial diversity remained unaffected by any of the variable proxies.

In aboveground compartments, host identity became dominant. In the leaf endosphere (Fig. 6E), bacterial community assembly was strongly structured by the plant’s species (estimate = -0.65, p = 0.001). Similarly, on the leaf surface (Fig. 6F), host identity also played a major role (estimate = -0.73, p = 0.001), with climatic variables exerting a marginal effect on bacterial β-diversity (estimate = -0.28, p = 0.08).

Altogether, these results enforce that both bacterial and fungal community assembly is governed by a shifting importance of environmental and host-related drivers along the soil–plant continuum; while abiotic filters dominate belowground, biotic ones become more prominent aboveground.

## Discussion and perspectives

The aim of our study was to provide a comprehensive overview of the diversity, recruitment strategies, and interconnections across microbial communities colonizing different soil–plant compartments of two endemic species from a pristine and rapidly warming sub-Antarctic ecosystem. Beyond the characterization of these microbiota, we sought to uncover how plant-intrinsic traits (phylogeny and architecture) and environmental factors (encompassing a wide range of abiotic variables) modulate their composition and connectivity. This comprehensive approach highlights the complexity of interactions between microbiota and their abiotic and biotic contexts. Going beyond previous compartment- or species-specific studies, we show that even in a low plant diversity and climatically constrained environment, microbial communities of co-occurring plant species, or even different organs of the same plant, respond differently to similar environmental cues. This was highlighted using different statistical approaches and the establishment of Structural Equation Models (SEMs) that hierarchize these complex interactions.

### Differential diversity and interconnections between soil–plant compartments

From a quantitative perspective, for the bacterial communities investigated across both belowground and aboveground compartments, we observed, for both plant species, a progressive decrease of diversity from soil to aerial tissues, a pattern that has been previously reported in various plant species [50–55]. This decline is generally interpreted as a consequence of multiple selective barriers that shape microbial communities along the soil–plant continuum since each soil–plant compartment is characterized by unique physicochemical conditions, immune defenses, and specific interfaces that act as environmental filters, selecting for a progressively narrower subset of microbial taxa [56], via recruitment from the outside environment or xylem transportation [57].However, we can not exclude that this difference across soil–plant compartments reflect distinct adaptations of microorganisms to reach and colonize plant tissues, which needs to be experimentally tested.

Although this soil to leaf diversity decay trend appears to be consistent for bacterial communities across plant species, our source-tracking analyses reveal plant species-specific differences in how adjacent compartments are interconnected suggesting different recruitment rulesbetween plant species. For instance, *P. kerguelensis* seems more permissive to bacterial exchange between roots and leaves, while in *P. antiscorbutica*, microbial transmission between belowground and aboveground parts appears more restricted. This finding contrasts with those reported by Schneijderberg et al. [58], for the annual Brassicaceae species *Arabidopsis thaliana* where a weaker rhizosphere effect was recorded, compared to long-lived co-occurring perennials. In our case, both studied plant species are perennial, meaning that rather than lifespan, phylogeny may play a significant role in microbial recruitment. Additionally, we can also hypothesize that plant architecture, such as the presence of a stem separating root and leaves in *P. antiscorbutica*, which is absent in *P. kerguelensis*, may play a role in regulating microbiota connectivity. While the reasons for such differences remain unclear, our observations argue in favor of setting up studies involving a large number of phylogenetically distinct plants with contrasting architectures, to assess the respective effects of plant phylogeny, physiology and architecture, not only on the diversity of associated microbiomes, but also on their modes of transmission and recruitment between organs of the same individual.

### Interplay between the host plant and environmental factors

Bacterial and fungal communities across all soil–plant compartments showed consistent responses to climatic variables, especially in aboveground compartments, albeit in species-specific ways. This finding is especially relevant for Kerguelen fellfields where harsh conditions (*e.g.* strong winds, high freeze–thaw cycles frequency) and therefore abiotic stress potentially impose strong selective pressures on microbial communities.

Along with climate, phosphorus and sulfur concentrations in soils, present at very low concentrations in this sub-Antarctic region (Table S1) [59], were in our study the most consistent edaphic predictors of microbial assembly and diversity across all soil–plant compartments. However, globally, soil bacterial diversity is mainly shaped by gradients in pH, moisture, and organic matter [60–63]. Similarly, for fungi, assembly and diversity is globally strongly influenced by pH [64–66]. These global studies typically include broader pH gradients, which may explain why pH was not identified as the main driver of diversity and assembly in Kerguelen’s fellfields, as it ranged from 5.87 to 6.84. In contrast, studies from other cold-climate ecosystems such as Antarctica have similarly identified phosphorus as one of the main drivers of soil fungal and bacterial diversity [67], along with air temperature which was found to be positively correlated with bacterial diversity [68]. However, beyond these prevailing drivers, compartment-level responses differed between the two plant species, suggesting that each host shapes its microbiota in a distinct way along the soil-to-shoot continuum.

In Kerguelen’s fellfields, nitrogen is one of the most limiting nutrients (Table S1) and emerged in our study as a key driver of both microbial α- and β-diversity across soil and plant compartments. Microorganisms implicated in the nitrogen-cycle are likely playing key roles in ecosystem functioning in fellfields and are probably of importance for plant’s fitness. Compared to bacteria, fungal diversity appeared more strongly shaped by host plant identity, suggesting that tight biotic interactions (*e.g.*, plant-fungal symbioses) play a dominant role. In phosphorus-limited sub-Antarctic soils, microbial organic matter mineralization and fungal symbioses are likely critical for plant phosphorus acquisition, as organic phosphorus must first be converted into bioavailable forms via microbial phosphatases or solubilized by plant-associated taxa. This role may be filled by dark septate endophytes (DSE), ubiquitous root-associated taxa that we found to dominate root-associated fungal communities in both studied plant species. DSE have indeed been found to increase phosphorus acquisition in alpine nutrients-poor environments [69] and are known to generally enhance plant tolerance to abiotic stress such as temperature, moisture and nutrient availability [70–73].

Interestingly, we found that leaf surface bacterial assembly was affected by soil properties, pointing to possibly direct connections between soil and leaf surface communities. This could arise from droplet’s splashing during rainfall or from wind-transported soil particles [74], supporting the idea that the leaf surface is at the junction between soil, air, and plant microbial pools [75, 76].

Finally, to comprehensively model and rank biotic and abiotic interactions on microbial communities, we used synthetic variables (derived from PCA axes, reducing environmental dimensions) to account for climatic and edaphic gradients and plant morphometric variability in SEMs. While it likely underestimates the strength of these environmental effects, the persistence of significant relationships despite this simplification highlights the robust influence of biotic and abiotic filters in shaping both fungal and bacterial community diversity, with varying intensity across soil–plant compartments.

## Conclusions

Our study underscores the specificity of plant–microbe interactions in pristine sub-Antarctic environments, where abiotic constraints, both climatic and edaphic, as well as host identity play pivotal roles in shaping bacterial and fungal community structure and diversity.

Ongoing climate change is likely to affect both abiotic conditions (*e.g.* temperature and precipitation regimes) and biotic components (*e.g.* changes in plant community composition and biological invasions). Thus, to predict the future trajectories of plant communities and their microbiomes, it is necessary to be able to anticipate differences in microbiome recruitment patterns and responses to stress factors between host species.

Moreover, if these microbiomes harbor a high proportion of endemic or geographically constrained taxa, particularly among dominant microbial groups, any disturbance, whether climatic, anthropogenic, or biotic (*e.g.*, invasions), may lead to disruption in biotic interactions.

Given the pace of climate change in these ecosystems, integrating long-term monitoring of the plant holobiont into existing biodiversity observations could provide early warnings of ecosystem functional shifts and help guide conservation strategies in the sub-Antarctic region.

Our findings advocate for extended sampling efforts of holobiont microbial biodiversity in these regions, which remain among the most understudied and ecologically vulnerable on the planet.

## Supplementary Information

Below is the link to the electronic supplementary material.

Supplementary Material 1

## Data Availability

Raw sequencing data from this study are available in the European Nucleotide Archive (EMBL-ENA) repository under accession numbers PRJEB89186 for 16S rRNA gene and PRJEB89188 for ITS.

## References

[1] Chialva M, Lanfranco L, Bonfante P. The plant microbiota: composition, functions, and engineering. Curr Opin Biotechnol. 2022;73:135–42. 10.1016/j.copbio.2021.07.003. 34392234 10.1016/j.copbio.2021.07.003

[2] Truyens S, Weyens N, Cuypers A, Vangronsveld J. Bacterial seed endophytes: genera, vertical transmission and interaction with plants: Bacterial seed endophytes. Environ Microbiol Rep. 2015;7:40–50. 10.1111/1758-2229.12181.

[3] Sánchez-Cañizares C, Jorrín B, Poole PS, Tkacz A. Understanding the holobiont: the interdependence of plants and their microbiome. Curr Opin Microbiol. 2017;38:188–96. 10.1016/j.mib.2017.07.001. 28732267 10.1016/j.mib.2017.07.001

[4] Mendes R, Garbeva P, Raaijmakers JM. The rhizosphere microbiome: significance of plant beneficial, plant pathogenic, and human pathogenic microorganisms. FEMS Microbiol Rev. 2013;37:634–63. 10.1111/1574-6976.12028. 23790204 10.1111/1574-6976.12028

[5] Jacoby R, Peukert M, Succurro A, Koprivova A, Kopriva S. The role of soil microorganisms in plant mineral nutrition—current knowledge and future directions. Front Plant Sci. 2017;8:1617. 10.3389/fpls.2017.01617. 28974956 10.3389/fpls.2017.01617 PMC5610682

[6] Stone BWG, Weingarten EA, Jackson CR. The Role of the Phyllosphere Microbiome in Plant Health and Function. In: Roberts JA, editor. Annual Plant Reviews online. 1st edition. Wiley; 2018. p. 533–56. 10.1002/9781119312994.apr0614.

[7] Koza N, Adedayo A, Babalola O, Kappo A. Microorganisms in plant growth and development: roles in abiotic stress tolerance and secondary metabolites secretion. Microorganisms. 2022;10:1528. 10.3390/microorganisms10081528. 36013946 10.3390/microorganisms10081528 PMC9415082

[8] Omae N, Tsuda K. Plant-microbiota interactions in abiotic stress environments. Mol Plant Microbe Interact. 2022;35:511–26. 10.1094/MPMI-11-21-0281-FI. 35322689 10.1094/MPMI-11-21-0281-FI

[9] Vandenkoornhuyse P, Quaiser A, Duhamel M, Le Van A, Dufresne A. The importance of the microbiome of the plant holobiont. New Phytol. 2015;206:1196–206. 10.1111/nph.13312. 25655016 10.1111/nph.13312

[10] Conrath U, Beckers GJM, Flors V, García-Agustín P, Jakab G, Mauch F, et al. Priming: Getting Ready for Battle. Mol Plant-Microbe Interactions®. 2006;19:1062–71. 10.1094/MPMI-19-1062. 10.1094/MPMI-19-1062 17022170

[11] Vannier N, Mony C, Bittebière A-K, Vandenkoornhuyse P. Epigenetic mechanisms and microbiota as a toolbox for plant phenotypic adjustment to environment. Front Plant Sci. 2015. 10.3389/fpls.2015.01159. 26779191 10.3389/fpls.2015.01159 PMC4688372

[12] Huitzil S, Sandoval-Motta S, Frank A, Aldana M. Modeling the role of the microbiome in evolution. Front Physiol. 2018;9:1836. 10.3389/fphys.2018.01836. 30618841 10.3389/fphys.2018.01836 PMC6307544

[13] Fitzpatrick CR, Copeland J, Wang PW, Guttman DS, Kotanen PM, Johnson MTJ. Assembly and ecological function of the root microbiome across angiosperm plant species. Proc Natl Acad Sci U S A. 2018. 10.1073/pnas.1717617115. 29358405 10.1073/pnas.1717617115 PMC5819437

[14] Zilber-Rosenberg I, Rosenberg E. Role of microorganisms in the evolution of animals and plants: the hologenome theory of evolution. FEMS Microbiol Rev. 2008;32:723–35. 10.1111/j.1574-6976.2008.00123.x. 18549407 10.1111/j.1574-6976.2008.00123.x

[15] Lebouvier M, Laparie M, Hullé M, Marais A, Cozic Y, Lalouette L, et al. The significance of the sub-Antarctic Kerguelen Islands for the assessment of the vulnerability of native communities to climate change, alien insect invasions and plant viruses. Biol Invasions. 2011;13:1195–208. 10.1007/s10530-011-9946-5.

[16] Lebouvier M, Frenot Y. Conservation and management in the French sub-Antarctic islands and surrounding seas. Pap Proc R Soc Tasman. 2007;:23–8. 10.26749/rstpp.141.1.23.

[17] Strullu D-G, Frenot Y, Maurice D, Gloaguen J-C, Plenchette C. Première contribution à l’étude des mycorhizes des îles Kerguelen. Comptes Rendus de l’Académie des Sciences. 1999;322:771–7. 10.1016/S0764-4469(00)80035-9.

[18] Pansu J, Winkworth RC, Hennion F, Gielly L, Taberlet P, Choler P. Long-lasting modification of soil fungal diversity associated with the introduction of rabbits to a remote sub-Antarctic archipelago. Biol Lett. 2015;11:20150408. 10.1098/rsbl.2015.0408. 26333663 10.1098/rsbl.2015.0408 PMC4614422

[19] Marchand LJ, Hennion F, Tarayre M, Martin M-C, Martins BR, Monard C. Fellfields of the Kerguelen Islands harbour specific soil microbiomes and rhizomicrobiomes of an endemic plant facing necrosis. Front Soil Sci. 2022;2:995716. 10.3389/fsoil.2022.995716.

[20] Lebre PH, Bosch J, Coclet C, Hallas R, Hogg ID, Johnson J, et al. Expanding Antarctic biogeography: microbial ecology of Antarctic island soils. Ecography. 2023;2023:e06568. 10.1111/ecog.06568.

[21] Aubert de la Rüe E. Observations sur les caractères et la répartition de la végétation des îles Kerguelen. Com Natl Fr Rech Antarct CNFRA-Biol. 1964;1:38.

[22] Van der Putten N, Verbruggen C, Ochyra R, Verleyen E, Frenot Y. Subantarctic flowering plants: pre-glacial survivors or post-glacial immigrants? J Biogeogr. 2010;37:582–92. 10.1111/j.1365-2699.2009.02217.x.

[23] Frenot Y, Lebouvier M, Gloaguen J-C, Hennion F, Vernon P, Chapuis J-L. Impact des changements climatiques et de la fréquentation humaine sur la biodiversité des îles subantarctiques françaises. Belgeo. 2006;:363–72. 10.4000/belgeo.12097.

[24] Verfaillie D, Favier V, Dumont M, Jomelli V, Gilbert A, Brunstein D, et al. Recent glacier decline in the Kerguelen Islands (49°S, 69°E) derived from modeling, field observations, and satellite data. J Geophys Res Earth Surf. 2015;120:637–54. 10.1002/2014JF003329.

[25] Nel W, Hedding DW, Rudolph EM. The sub-Antarctic islands are increasingly warming in the 21st century. Antarct Sci. 2023;35:124–6. 10.1017/S0954102023000056.

[26] le Roux PC, McGeoch MA. Rapid range expansion and community reorganization in response to warming. Glob Change Biol. 2008;14:2950–62. 10.1111/j.1365-2486.2008.01687.x.

[27] Daly EZ, Gerlich HS, Frenot Y, Høye TT, Holmstrup M, Renault D. Climate change helps polar invasives establish and flourish: evidence from long-term monitoring of the blowfly *Calliphora vicina*. Biology. 2023;12:111. 10.3390/biology12010111. 36671803 10.3390/biology12010111 PMC9856047

[28] Leihy RI, Duffy GA, Nortje E, Chown SL. High resolution temperature data for ecological research and management on the Southern Ocean Islands. Sci Data. 2018;5:180177. 10.1038/sdata.2018.177. 30179229 10.1038/sdata.2018.177 PMC6122169

[29] Frenot Y, Gloaguen JC, Massé L, Lebouvier M. Human activities, ecosystem disturbance and plant invasions in subantarctic Crozet, Kerguelen and Amsterdam Islands. Biol Conserv. 2001;101:33–50. 10.1016/S0006-3207(01)00052-0.

[30] Frenot Y, Chown SL, Whinam J, Selkirk PM, Convey P, Skotnicki M, et al. Biological invasions in the Antarctic: extent, impacts and implications. Biol Rev. 2005;80:45–72. 10.1017/S1464793104006542. 15727038 10.1017/s1464793104006542

[31] Wild J, Kopecký M, Macek M, Šanda M, Jankovec J, Haase T. Climate at ecologically relevant scales: a new temperature and soil moisture logger for long-term microclimate measurement. Agric For Meteorol. 2019;268:40–7. 10.1016/j.agrformet.2018.12.018.

[32] Wemheuer B, Wemheuer F. Assessing Bacterial and Fungal Diversity in the Plant Endosphere. In: Streit WR, Daniel R, editors. Metagenomics. New York, NY: Springer New York; 2017. p. 75–84. 10.1007/978-1-4939-6691-2_6. 10.1007/978-1-4939-6691-2_6 27900685

[33] Walker FM, Williamson CHD, Sanchez DE, Sobek CJ, Chambers CL. Species from feces: order-wide identification of Chiroptera from guano and other non-invasive genetic samples. PLoS ONE. 2016;11:e0162342. 10.1371/journal.pone.0162342. 27654850 10.1371/journal.pone.0162342 PMC5031397

[34] Caporaso JG, Lauber CL, Walters WA, Berg-Lyons D, Huntley J, Fierer N, et al. Ultra-high-throughput microbial community analysis on the Illumina HiSeq and MiSeq platforms. ISME J. 2012;6:1621–4. 10.1038/ismej.2012.8. 22402401 10.1038/ismej.2012.8 PMC3400413

[35] Apprill A, McNally S, Parsons R, Weber L. Minor revision to V4 region SSU rRNA 806R gene primer greatly increases detection of SAR11 bacterioplankton. Aquat Microb Ecol. 2015;75:129–37. 10.3354/ame01753.

[36] Thompson LR, Sanders JG, McDonald D, Amir A, Ladau J, Locey KJ, et al. A communal catalogue reveals Earth’s multiscale microbial diversity. Nature. 2017;551:457–63. 10.1038/nature24621. 29088705 10.1038/nature24621 PMC6192678

[37] Ihrmark K, Bödeker ITM, Cruz-Martinez K, Friberg H, Kubartova A, Schenck J, et al. New primers to amplify the fungal ITS2 region - evaluation by 454-sequencing of artificial and natural communities. FEMS Microbiol Ecol. 2012;82:666–77. 10.1111/j.1574-6941.2012.01437.x. 22738186 10.1111/j.1574-6941.2012.01437.x

[38] Martin M. Cutadapt removes adapter sequences from high-throughput sequencing reads. EMBnet J. 2011;17:10–2. 10.14806/ej.17.1.200.

[39] Callahan BJ, McMurdie PJ, Rosen MJ, Han AW, Johnson AJA, Holmes SP. DADA2: high-resolution sample inference from Illumina amplicon data. Nat Methods. 2016;13:581–3. 10.1038/nmeth.3869. 27214047 10.1038/nmeth.3869 PMC4927377

[40] Katoh K, Standley DM. MAFFT multiple sequence alignment software version 7: improvements in performance and usability. Mol Biol Evol. 2013;30:772–80. 10.1093/molbev/mst010. 23329690 10.1093/molbev/mst010 PMC3603318

[41] Price MN, Dehal PS, Arkin AP. Fasttree 2 – approximately maximum-likelihood trees for large alignments. PLoS ONE. 2010;5:e9490. 10.1371/journal.pone.0009490. 20224823 10.1371/journal.pone.0009490 PMC2835736

[42] Wright ES, Yilmaz LS, Noguera DR. DECIPHER, a search-based approach to chimera identification for 16S rRNA sequences. Appl Environ Microbiol. 2012;78:717–25. 10.1128/AEM.06516-11. 22101057 10.1128/AEM.06516-11 PMC3264099

[43] Quast C, Pruesse E, Yilmaz P, Gerken J, Schweer T, Yarza P, et al. The SILVA ribosomal RNA gene database project: improved data processing and web-based tools. Nucleic Acids Res. 2012;41:D590–6. 10.1093/nar/gks1219. 23193283 10.1093/nar/gks1219 PMC3531112

[44] Abarenkov K, Zirk A, Piirmann T, Pöhönen R, Ivanov F, Nilsson HR, et al. UNITE general FASTA release for eukaryotes 2. Version 04.04.2024. UNITE Community. 2024. 10.15156/BIO/2959335.

[45] McKnight DT, Huerlimann R, Bower DS, Schwarzkopf L, Alford RA, Zenger KR. MicroDecon: a highly accurate read-subtraction tool for the post-sequencing removal of contamination in metabarcoding studies. Environ DNA. 2019;1:14–25. 10.1002/edn3.11.

[46] Gloor GB, Macklaim JM, Pawlowsky-Glahn V, Egozcue JJ. Microbiome datasets are compositional: and this is not optional. Front Microbiol. 2017;8:2224. 10.3389/fmicb.2017.02224. 29187837 10.3389/fmicb.2017.02224 PMC5695134

[47] Quinn TP, Erb I, Richardson MF, Crowley TM. Understanding sequencing data as compositions: an outlook and review. Bioinformatics. 2018;34:2870–8. 10.1093/bioinformatics/bty175. 29608657 10.1093/bioinformatics/bty175 PMC6084572

[48] Knights D, Kuczynski J, Charlson ES, Zaneveld J, Mozer MC, Collman RG, et al. Bayesian community-wide culture-independent microbial source tracking. Nat Methods. 2011;8:761–3. 10.1038/nmeth.1650. 21765408 10.1038/nmeth.1650 PMC3791591

[49] Lefcheck JS. PiecewiseSEM : piecewise structural equation modelling in r for ecology, evolution, and systematics. Methods Ecol Evol. 2016;7:573–9. 10.1111/2041-210X.12512.

[50] Bernard J, Wall CB, Costantini MS, Rollins RL, Atkins ML, Cabrera FP, et al. Plant part and a steep environmental gradient predict plant microbial composition in a tropical watershed. ISME J. 2021;15:999–1009. 10.1038/s41396-020-00826-5. 33188299 10.1038/s41396-020-00826-5 PMC8115680

[51] Xiong C, Zhu Y, Wang J, Singh B, Han L, Shen J, et al. Host selection shapes crop microbiome assembly and network complexity. New Phytol. 2021;229:1091–104. 10.1111/nph.16890. 32852792 10.1111/nph.16890

[52] Siegenthaler A, Skidmore AK, De Groot GA, Laros I, Rousseau M, Duan Y. Temperate tree microbiomes: divergent soil and phyllosphere microbial communities share few but dominant taxa. Plant Soil. 2024;496:319–40. 10.1007/s11104-023-06364-1.

[53] Wei G, Ning K, Zhang G, Yu H, Yang S, Dai F, et al. Compartment niche shapes the assembly and network of *Cannabis sativa*-associated microbiome. Front Microbiol. 2021;12:714993. 10.3389/fmicb.2021.714993. 34675893 10.3389/fmicb.2021.714993 PMC8524047

[54] Coleman-Derr D, Desgarennes D, Fonseca-Garcia C, Gross S, Clingenpeel S, Woyke T, et al. Plant compartment and biogeography affect microbiome composition in cultivated and native *Agave* species. New Phytol. 2015;209:798–11. 10.1111/nph.13697. 26467257 10.1111/nph.13697 PMC5057366

[55] Swift JF, Migicovsky Z, Trello GE, Miller AJ. Grapevine bacterial communities display compartment-specific dynamics over space and time within the Central Valley of California. Environ Microbiome. 2023;18:84. 10.1186/s40793-023-00539-0. 37996903 10.1186/s40793-023-00539-0 PMC10668525

[56] Fitzpatrick CR, Salas-González I, Conway JM, Finkel OM, Gilbert S, Russ D, et al. The plant microbiome: from ecology to reductionism and beyond. Annu Rev Microbiol. 2020;74:81–100. 10.1146/annurev-micro-022620-014327. 32530732 10.1146/annurev-micro-022620-014327

[57] Compant S, Samad A, Faist H, Sessitsch A. A review on the plant microbiome: ecology, functions, and emerging trends in microbial application. J Adv Res. 2019;19:29–37. 10.1016/j.jare.2019.03.004. 31341667 10.1016/j.jare.2019.03.004 PMC6630030

[58] Schneijderberg M, Cheng X, Franken C, De Hollander M, Van Velzen R, Schmitz L, et al. Quantitative comparison between the rhizosphere effect of *Arabidopsis thaliana* and co-occurring plant species with a longer life history. ISME J. 2020;14:2433–48. 10.1038/s41396-020-0695-2. 32641729 10.1038/s41396-020-0695-2 PMC7490400

[59] McDowell RW, Noble A, Pletnyakov P, Haygarth PM. A global database of soil plant available phosphorus. Sci Data. 2023;10:125. 10.1038/s41597-023-02022-4. 36882412 10.1038/s41597-023-02022-4 PMC9992394

[60] Kaiser K, Wemheuer B, Korolkow V, Wemheuer F, Nacke H, Schöning I, et al. Driving forces of soil bacterial community structure, diversity, and function in temperate grasslands and forests. Sci Rep. 2016;6:33696. 10.1038/srep33696. 27650273 10.1038/srep33696 PMC5030646

[61] Bahram M, Hildebrand F, Forslund SK, Anderson JL, Soudzilovskaia NA, Bodegom PM, et al. Structure and function of the global topsoil microbiome. Nature. 2018;560:233–7. 10.1038/s41586-018-0386-6. 30069051 10.1038/s41586-018-0386-6

[62] Bastida F, Eldridge DJ, García C, Kenny Png G, Bardgett RD, Delgado-Baquerizo M. Soil microbial diversity–biomass relationships are driven by soil carbon content across global biomes. ISME J. 2021;15:2081–91. 10.1038/s41396-021-00906-0. 33564112 10.1038/s41396-021-00906-0 PMC8245509

[63] Labouyrie M, Ballabio C, Romero F, Panagos P, Jones A, Schmid MW, et al. Patterns in soil microbial diversity across Europe. Nat Commun. 2023;14:3311. 10.1038/s41467-023-37937-4. 37291086 10.1038/s41467-023-37937-4 PMC10250377

[64] Tedersoo L, Bahram M, Põlme S, Kõljalg U, Yorou NS, Wijesundera R, et al. Global diversity and geography of soil fungi. Science. 2014;346:1256688. 10.1126/science.1256688. 25430773 10.1126/science.1256688

[65] George PBL, Lallias D, Creer S, Seaton FM, Kenny JG, Eccles RM, et al. Divergent national-scale trends of microbial and animal biodiversity revealed across diverse temperate soil ecosystems. Nat Commun. 2019;10:1107. 10.1038/s41467-019-09031-1. 30846683 10.1038/s41467-019-09031-1 PMC6405921

[66] Djemiel C, Dequiedt S, Horrigue W, Bailly A, Lelièvre M, Tripied J, et al. Unraveling biogeographical patterns and environmental drivers of soil fungal diversity at the French national scale. SOIL. 2024;10:251–73. 10.5194/soil-10-251-2024.

[67] Siciliano SD, Palmer AS, Winsley T, Lamb E, Bissett A, Brown MV, et al. Soil fertility is associated with fungal and bacterial richness, whereas pH is associated with community composition in polar soil microbial communities. Soil Biol Biochem. 2014;78:10–20. 10.1016/j.soilbio.2014.07.005.

[68] Dennis PG, Newsham KK, Rushton SP, O’Donnell AG, Hopkins DW. Soil bacterial diversity is positively associated with air temperature in the maritime Antarctic. Sci Rep. 2019;9:2686. 10.1038/s41598-019-39521-7. 30804443 10.1038/s41598-019-39521-7 PMC6389919

[69] Almario J, Jeena G, Wunder J, Langen G, Zuccaro A, Coupland G, et al. Root-associated fungal microbiota of nonmycorrhizal *Arabis alpina* and its contribution to plant phosphorus nutrition. Proc Natl Acad Sci. 2017;114. 10.1073/pnas.1710455114. 10.1073/pnas.1710455114 PMC5676915 28973917

[70] Hill PW, Broughton R, Bougoure J, Havelange W, Newsham KK, Grant H, et al. Angiosperm symbioses with non-mycorrhizal fungal partners enhance N acquisition from ancient organic matter in a warming maritime Antarctic. Ecol Lett. 2019;22:2111–9. 10.1111/ele.13399. 31621153 10.1111/ele.13399 PMC6899649

[71] Jumpponen A, Trappe JM. Dark septate endophytes: a review of facultative biotrophic root-colonizing fungi. New Phytol. 1998;140:295–10. 10.1046/j.1469-8137.1998.00265.x. 33862835 10.1046/j.1469-8137.1998.00265.x

[72] Malicka M, Magurno F, Piotrowska-Seget Z. Plant association with dark septate endophytes: when the going gets tough (and stressful), the tough fungi get going. Chemosphere. 2022;302:134830. 10.1016/j.chemosphere.2022.134830. 35525444 10.1016/j.chemosphere.2022.134830

[73] Newsham KK, Upson R, Read DJ. Mycorrhizas and dark septate root endophytes in polar regions. Fungal Ecol. 2009;2:10–20. 10.1016/j.funeco.2008.10.005. 10.1007/s00572-009-0260-3 19495811

[74] Ossola R, Farmer D. The chemical landscape of leaf surfaces and its interaction with the atmosphere. Chem Rev. 2024;124:5764–94. 10.1021/acs.chemrev.3c00763. 38652704 10.1021/acs.chemrev.3c00763 PMC11082906

[75] Vacher C, Hampe A, Porté AJ, Sauer U, Compant S, Morris CE. The phyllosphere: microbial jungle at the plant-climate interface. Annu Rev Ecol Evol Syst. 2016;47:1–24. 10.1146/annurev-ecolsys-121415-032238.

[76] Smets W, Chock MK, Walsh CM, Vanderburgh CQ, Kau E, Lindow SE, et al. Leaf side determines the relative importance of dispersal versus host filtering in the phyllosphere microbiome. mBio. 2023;:e01111–23. 10.1128/mbio.01111-23. 10.1128/mbio.01111-23 PMC10470611 37436063

