# Supplementary material for: Interplay between host and environmental filters drives plant-associated microbiomes in the remote sub-Antarctic Kerguelen Islands
Source: Environ Microbiome. 2025 Dec 22;20:154. doi: 10.1186/s40793-025-00814-2 (PMC12723888; doi:10.1186/s40793-025-00814-2)
Supplement: Supplementary file 1 — Supplementary Material 1 [file 40793_2025_814_MOESM1_ESM.docx]

**Interplay between host and environmental filters drives plant-associated microbiomes in remote the sub-Antarctic Kerguelen Islands**

Constance Bertrand^1^*, Roland Marmeisse^2^*, Marie-Claire Martin^1^, Françoise Binet^1^*

^1^ Univ Rennes, CNRS, ECOBIO [(Ecosystèmes, biodiversité, évolution)] - UMR 6553, F-35000 Rennes, France

^2^ Institut de Systématique, Evolution, Biodiversité (ISYEB), Muséum National d’Histoire Naturelle, CNRS, Sorbonne Université, EPHE, Université des Antilles, 57 Rue Cuvier, CP39, 75005 Paris, France

**Corresponding authors:**

*constance.bertrand@univ-rennes.fr

*roland.marmeisse@mnhn.fr

*francoise.binet@univ-rennes.fr

**Supplementary figures**


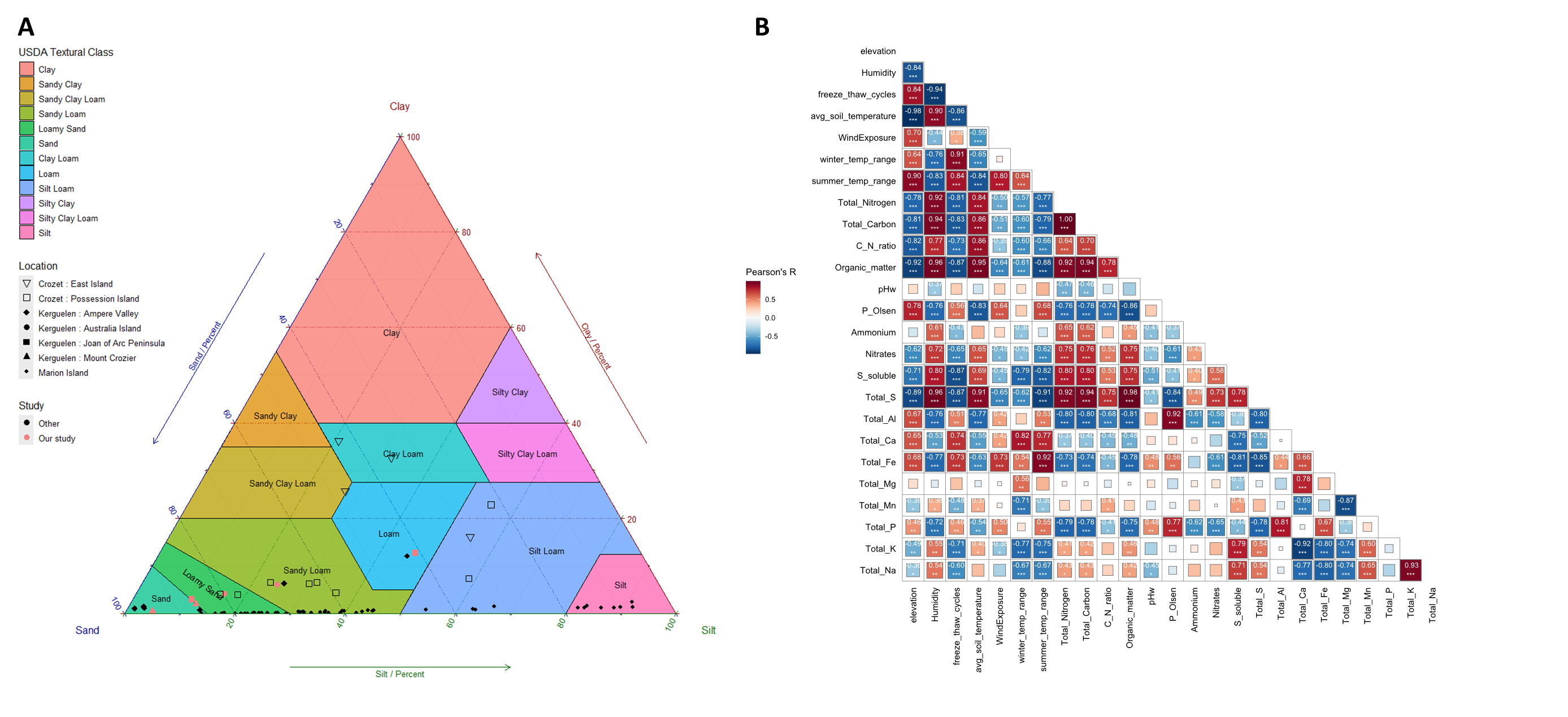


**Fig. S1 Soil texture and environmental variable correlations.** USDA texture diagram comparing Kerguelen fellfields’ bulk soils sampled in this study (red dots) with those from other sub-Antarctic islands of the South Indian Ocean Province (black dots) **(A)** [1–3]. Pearson correlation matrix highlighting relationships among all measured climatic and edaphic soil properties in the sampled Kerguelen plots **(B)**.


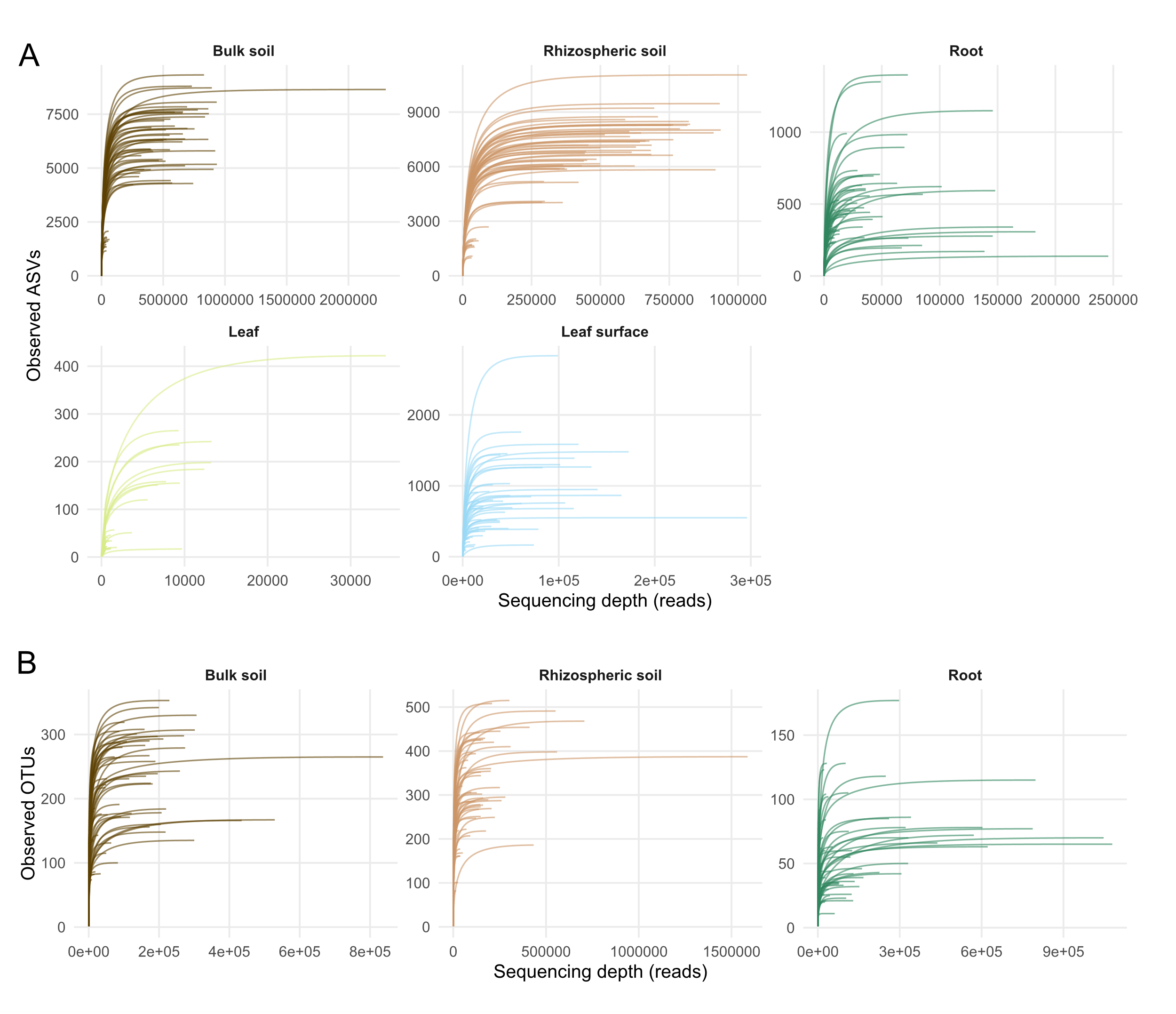


**Fig. S2 Rarefaction curves for the bacterial (A) and fungal (B) datasets across soil-plant compartments**


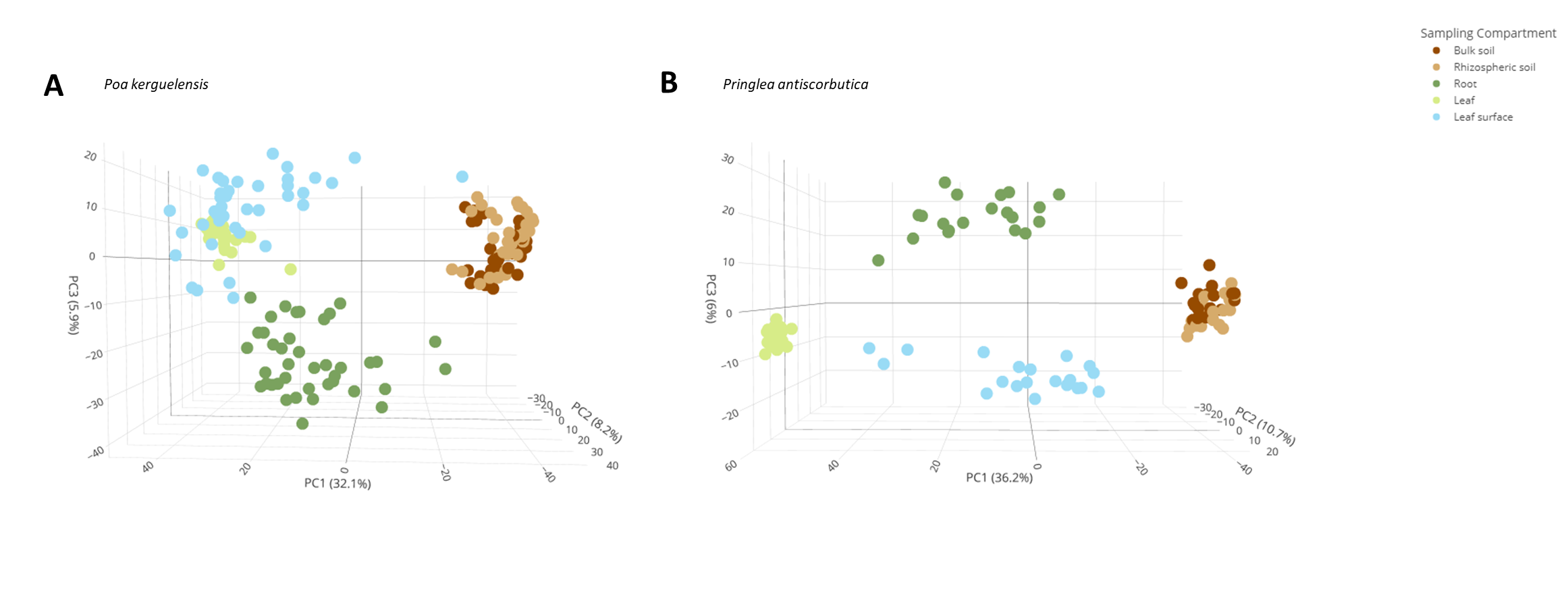


**Fig. S3 Three dimensional PCoAs highlighting the contribution of PC3 in distinguishing leaf surface and endophytic bacterial communities in *Poa kerguelensis* (A) and *Pringlea antiscorbutica* (B).** See Fig. 2 in the main text for the detailed legend.


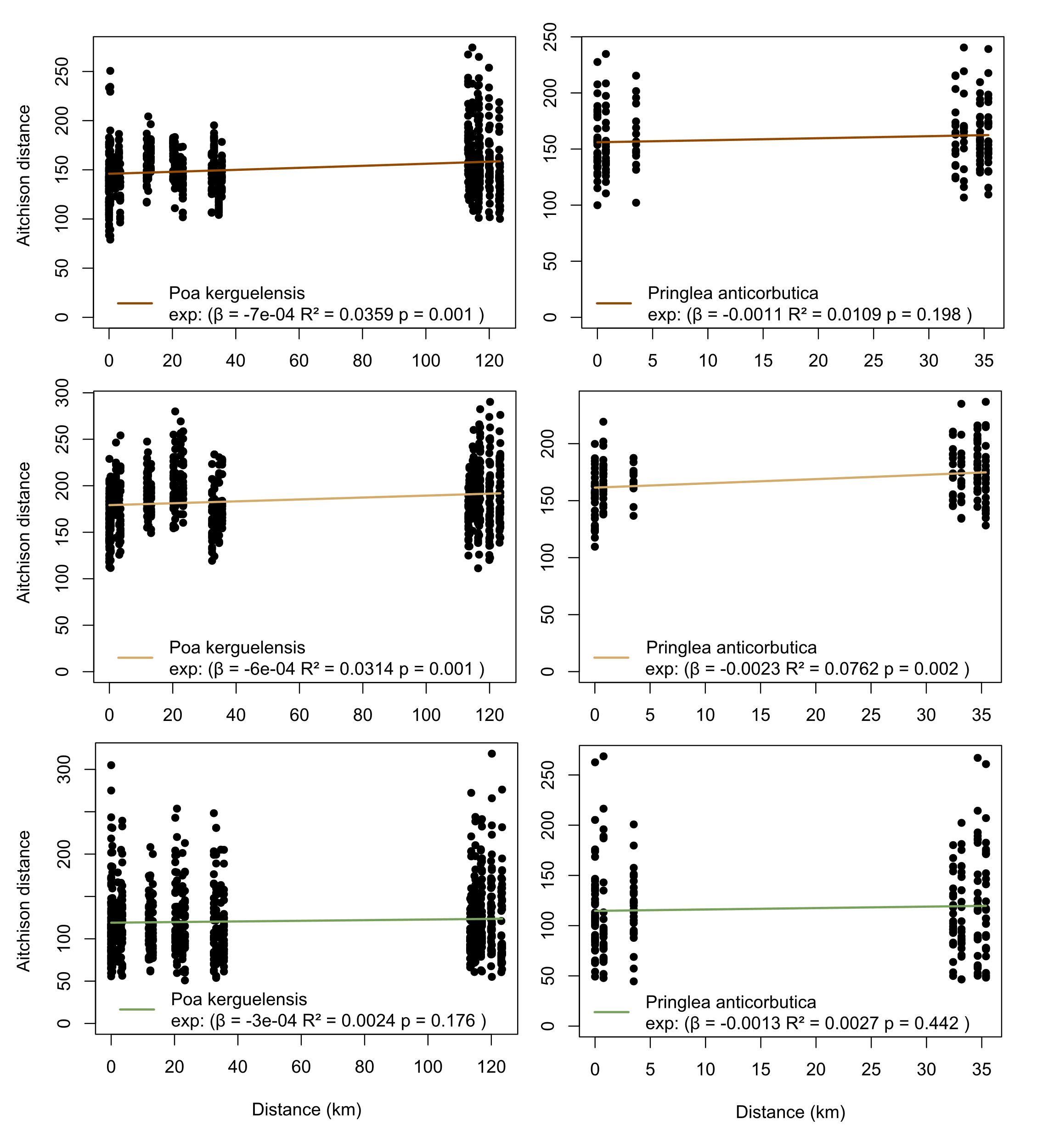


**Fig. S4 Fungal community distance-decay relationships across compartments and host species.** Fungal distance-decay relationships are modeled as a negative exponential function on Aitchison distance for each soil-plant compartment (from top to bottom: bulk soil, rhizospheric soil and roots) and for each host plant species (*P. kerguelensis* on the left and *P. antiscorbutica* on the right).


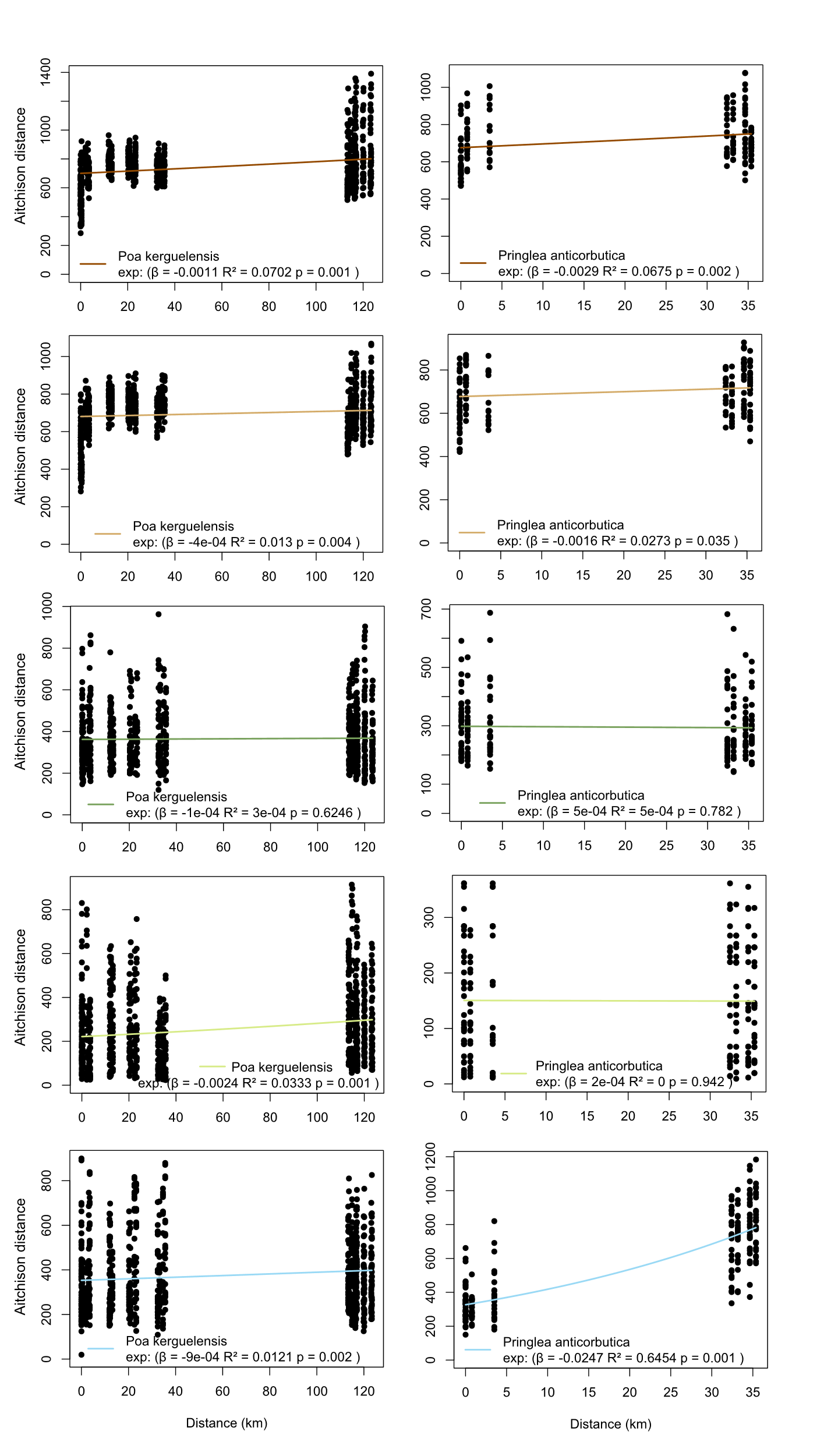


**Fig. S5 Bacterial community distance-decay relationships across compartments and host species.** Bacterial distance-decay relationships modeled as a negative exponential function on Aitchison distance for each soil-plant compartment (from top to bottom: bulk soil, rhizospheric soil, roots, leaf endosphere and leaf surface) and for each host plant species (*P. kerguelensis* on the left and *P. antiscorbutica* on the right).

**Supplementary tables**

**Tab. S1 Location and abiotic parameters characterizing each of the sampled plots (mean ± sd when replicated).** Sites: AUS, Australia Island; PJA, Joan of Arc Peninsula; CRO, Mount Crozier; PCH, Port-Christmas. Plot elevation: “H” indicates a high-altitude plot, “L” indicates a low-altitude plot. AT the elevated PJA site, the two plant species did not co-occur in the same plot, a “1” or “2” was added after the elevation “H” marker to distinguish plant plots.


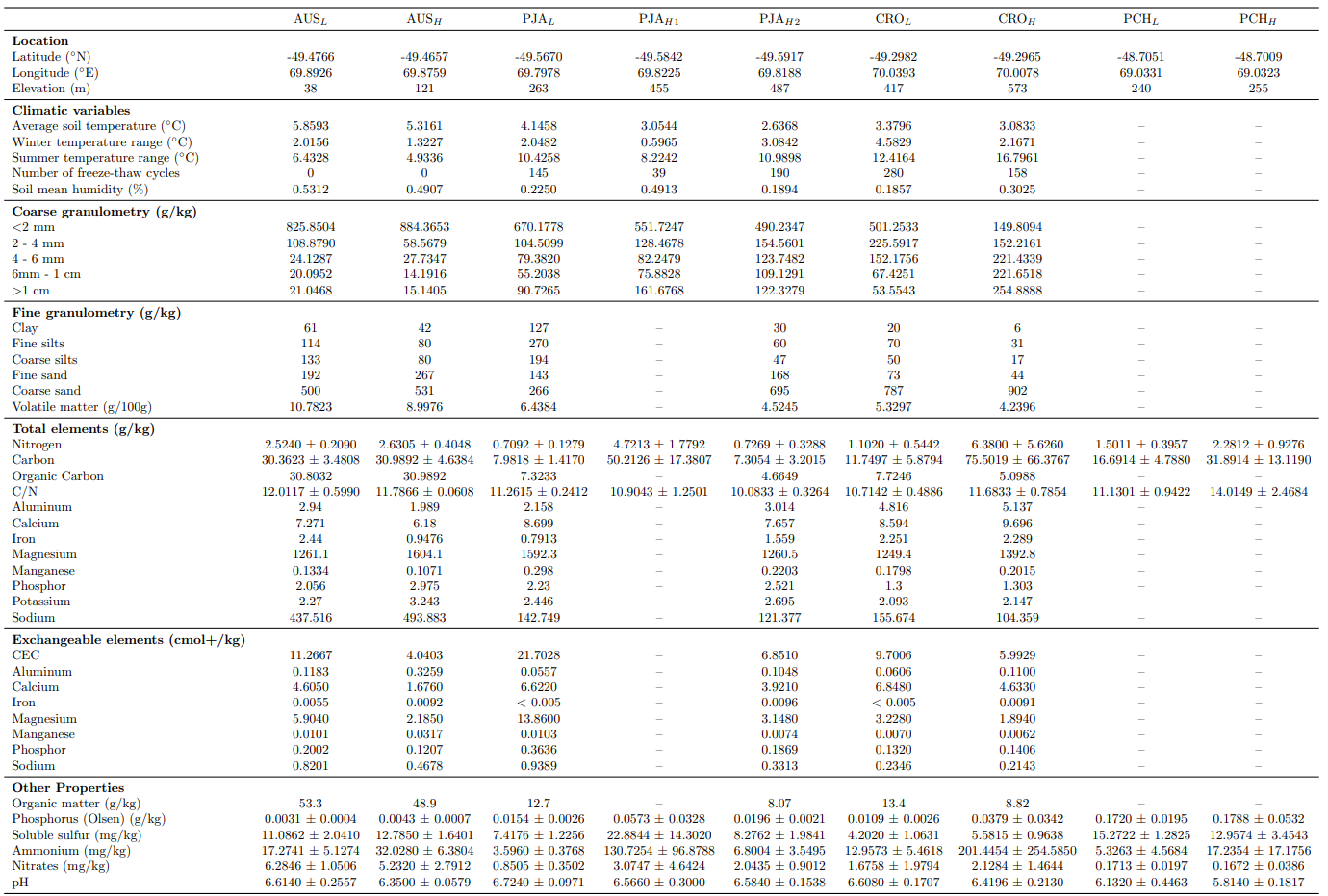


**Tab S2**  **Morphological and reproductive traits measured on sampled plants.** Values are presented as mean ± standard deviation, with minimum and maximum values in parentheses. Units are reported in the “Units” column and sample size (n) is given for each plant species.

| **Morphological Trait** | **Units** | ***Poa kerguelensis* (n=40)** | ***Pringlea antiscorbutica* (n=20)** |
| --- | --- | --- | --- |
| Height | *cm* | 3.60 ± 0.86 (2.00 – 6.50) | 6.97 ± 2.18 (3.50 – 11.50) |
| Largest diameter | *cm* | 6.83 ± 2.59 (3.00 – 11.00) | 9.88 ± 2.86 (5.50 – 16.00) |
| Orthogonal diameter | *cm* | 5.08 ± 1.49 (2.50 – 9.00) | 8.93 ± 2.52 (5.00 – 15.50) |
| Length of the largest leaf (with petiole) | *cm* | – | 7.43 ± 2.02 (4.90 – 11.80) |
| Length of the largest leaf (without petiole) | *cm* | – | 4.16 ± 0.82 (2.90 – 5.70) |
| Width of the largest leaf | *cm* | – | 3.68 ± 0.77 (1.80 – 4.60) |
| No. of inflorescences | */* | 28.17 ± 30.08 (1 – 147) | – |
| No. of leaves | */* | 57.69 ± 20.52 (21 – 120) | 21.71 ± 6.44 (11 – 36) |
| Maximum root diameter | *cm* | – | 1.23 ± 0.39 (0.63 – 1.81) |
| Diameter at collar | *cm* | – | 1.14 ± 0.34 (0.61 – 1.61) |
| Dry shoot weight | *g* | 2.88 ± 2.28 (0.08 – 8.19) | – |
| Dry root weight | *g* | 1.10 ± 1.37 (0.07 – 6.99) | – |

**Tab. S3 Pairwise comparison between soil-plant compartments community assembly (calculated as Aitchison distance) calculated with PERMANOVAs.**

|  | **Bacteria** | | | | **Fungi** | | | | |
| --- | --- | --- | --- | --- | --- | --- | --- | --- | --- |
|  | **df** | **F** | **R²** | **p** | | **df** | **F** | **R²** | **p** |
| **Poa kerguelensis** |  |  |  |  | |  |  |  |  |
| Root vs Leaf | 1 | 7.2920 | 0.0855 | 0.01 | |  |  |  |  |
| Root vs Rhizospheric soil | 1 | 13.0171 | 0.1430 | 0.01 | | 1 | 9.2051 | 0.1056 | 0.003 |
| Root vs Bulk soil | 1 | 12.6424 | 0.1410 | 0.01 | | 1 | 9.0185 | 0.1048 | 0.003 |
| Root vs Leaf surface | 1 | 7.7584 | 0.0905 | 0.01 | |  |  |  |  |
| Leaf vs Rhizospheric soil | 1 | 16.4080 | 0.1738 | 0.01 | |  |  |  |  |
| Leaf vs Bulk soil | 1 | 16.0684 | 0.1727 | 0.01 | |  |  |  |  |
| Leaf vs Leaf surface | 1 | 7.1455 | 0.0839 | 0.01 | |  |  |  |  |
| Rhizospheric soil vs Bulk soil | 1 | 1.6995 | 0.0216 | 0.37 | | 1 | 1.7652 | 0.0224 | 0.060 |
| Rhizospheric soil vs Leaf surface | 1 | 14.5443 | 0.1572 | 0.01 | |  |  |  |  |
| Bulk soil vs Leaf surface | 1 | 14.4022 | 0.1576 | 0.01 | |  |  |  |  |
| **Pringlea antiscorbutica** |  |  |  |  | |  |  |  |  |
| Root vs Leaf | 1 | 7.6346 | 0.1673 | 0.01 | |  |  |  |  |
| Root vs Rhizospheric soil | 1 | 13.6387 | 0.2748 | 0.01 | | 1 | 8.8997 | 0.1982 | 0.003 |
| Root vs Bulk soil | 1 | 13.5158 | 0.2730 | 0.01 | | 1 | 8.1672 | 0.1849 | 0.003 |
| Root vs Leaf surface | 1 | 3.6667 | 0.0880 | 0.01 | |  |  |  |  |
| Leaf vs Rhizospheric soil | 1 | 17.7148 | 0.3298 | 0.01 | |  |  |  |  |
| Leaf vs Bulk soil | 1 | 17.5054 | 0.3272 | 0.01 | |  |  |  |  |
| Leaf vs Leaf surface | 1 | 4.1741 | 0.0990 | 0.01 | |  |  |  |  |
| Rhizospheric soil vs Bulk soil | 1 | 0.9944 | 0.0284 | 1.00 | | 1 | 1.9950 | 0.0554 | 0.015 |
| Rhizospheric soil vs Leaf surface | 1 | 10.5968 | 0.2274 | 0.01 | |  |  |  |  |
| Bulk soil vs Leaf surface | 1 | 10.6820 | 0.2288 | 0.01 | |  |  |  |  |

**Tab. S4 Root most abundant molecular taxa in *P. kerguelensis*.** Root specificity corresponds to the abundance in which a given taxon was detected in the root compartment relative to its total abundance across all soil-plant compartments, reflecting its preferential association with endophytic root tissues.

| **Bacterial ASVs and fungal OTUs** | **Root specificity** | **Relative abundance in roots** | **Kingdom** | **Phylum** | **Class** | **Order** | **Family** | **Genus** | **Species** |
| --- | --- | --- | --- | --- | --- | --- | --- | --- | --- |
| ASV_000022 | 0.9961 | 0.1339 | Bacteria | Proteobacteria | Gammaproteobacteria | Pseudomonadales | Pseudomonadaceae | Pseudomonas |  |
| ASV_000027 | 0.8638 | 0.0545 | Bacteria | Proteobacteria | Gammaproteobacteria | Pseudomonadales | Pseudomonadaceae | Pseudomonas |  |
| ASV_000036 | 0.9451 | 0.0628 | Bacteria | Proteobacteria | Gammaproteobacteria | Pseudomonadales | Pseudomonadaceae | Pseudomonas |  |
| ASV_000042 | 0.9992 | 0.0393 | Bacteria | Proteobacteria | Gammaproteobacteria | Pseudomonadales | Pseudomonadaceae | Pseudomonas |  |
| ASV_000051 | 0.5921 | 0.0271 | Bacteria | Proteobacteria | Gammaproteobacteria | Burkholderiales | Oxalobacteraceae | Duganella | zoogloeoides |
| ASV_000063 | 0.9872 | 0.0428 | Bacteria | Proteobacteria | Gammaproteobacteria | Pseudomonadales | Pseudomonadaceae | Pseudomonas |  |
| ASV_000086 | 0.7848 | 0.0140 | Bacteria | Proteobacteria | Gammaproteobacteria | Burkholderiales | Oxalobacteraceae | Janthinobacterium |  |
| ASV_000153 | 0.9925 | 0.0428 | Bacteria | Chloroflexi | Ktedonobacteria | Ktedonobacterales | Ktedonobacteraceae |  |  |
| ASV_000169 | 0.8386 | 0.0116 | Bacteria | Proteobacteria | Gammaproteobacteria | Enterobacterales | Morganellaceae | Buchnera |  |
| ASV_000217 | 0.7862 | 0.0122 | Bacteria | Proteobacteria | Gammaproteobacteria | Enterobacterales | Enterobacteriaceae | Klebsiella |  |
| ASV_000237 | 0.7983 | 0.0115 | Bacteria | Proteobacteria | Gammaproteobacteria | Enterobacterales | Enterobacteriaceae | Escherichia-Shigella |  |
| ASV_000253 | 0.8719 | 0.0178 | Bacteria | Bacteroidota | Bacteroidia | Sphingobacteriales | Sphingobacteriaceae | Pedobacter |  |
| ASV_000297 | 0.6657 | 0.0106 | Bacteria | Proteobacteria | Gammaproteobacteria | Burkholderiales | Oxalobacteraceae | Duganella |  |
| ASV_000329 | 1.0000 | 0.0212 | Bacteria | Actinobacteriota | Actinobacteria | Pseudonocardiales | Pseudonocardiaceae | Umezawaea |  |
| OTU_2 | 1.0000 | 0.3103 | Fungi | Ascomycota | Dothideomycetes | Pleosporales | Phaeosphaeriaceae | Ophiosphaerella |  |
| OTU_5 | 1.0000 | 0.2108 | Fungi | Ascomycota | Leotiomycetes |  |  |  |  |
| OTU_9 | 1.0000 | 0.0755 | Fungi | Ascomycota | Dothideomycetes | Pleosporales | Lindgomycetaceae | Clohesyomyces |  |
| OTU_16 | 1.0000 | 0.0685 | Fungi | Ascomycota | Dothideomycetes | Pleosporales |  |  |  |
| OTU_34 | 0.7373 | 0.0355 | Fungi | Ascomycota | Sordariomycetes | Sordariomycetes_ord_Incertae_sedis | Junewangiaceae | Junewangia |  |
| OTU_58 | 0.5289 | 0.0218 | Fungi | Ascomycota | Leotiomycetes | Helotiales | Helotiales_fam_Incertae_sedis |  |  |
| OTU_68 | 1.0000 | 0.0372 | Fungi | Ascomycota | Leotiomycetes | Helotiales | Pezizellaceae | Microscypha |  |
| OTU_90 | 0.8048 | 0.0296 | Fungi | Ascomycota | Sordariomycetes |  |  |  |  |
| OTU_108 | 0.8708 | 0.0119 | Fungi | Ascomycota | Sordariomycetes |  |  |  |  |
| OTU_134 | 0.9891 | 0.0121 | Fungi | Ascomycota | Dothideomycetes | Pleosporales | Melanommataceae |  |  |
| OTU_151 | 0.9574 | 0.0118 | Fungi | Ascomycota | Leotiomycetes | Helotiales |  |  |  |

**Tab. S5 Root most abundant molecular taxa in *P. antiscorbutica*.** Root specificity corresponds to the abundance in which a given taxon was detected in the root compartment relative to its total abundance across all soil-plant compartments, reflecting its preferential association with endophytic root tissues.

| **Bacterial ASVs and fungal OTUs** | **Root specificity** | **Relative abundance in roots** | **Kindom** | **Phylum** | **Class** | **Order** | **Family** | **Genus** | **Species** |
| --- | --- | --- | --- | --- | --- | --- | --- | --- | --- |
| ASV_000022 | 0.7198 | 0.0164 | Bacteria | Proteobacteria | Gammaproteobacteria | Pseudomonadales | Pseudomonadaceae | Pseudomonas |  |
| ASV_000031 | 0.7093 | 0.1249 | Bacteria | Firmicutes | Clostridia | Clostridiales | Clostridiaceae | Clostridium sensu stricto 1 |  |
| ASV_000036 | 0.5882 | 0.0333 | Bacteria | Proteobacteria | Gammaproteobacteria | Pseudomonadales | Pseudomonadaceae | Pseudomonas |  |
| ASV_000042 | 0.9335 | 0.0252 | Bacteria | Proteobacteria | Gammaproteobacteria | Pseudomonadales | Pseudomonadaceae | Pseudomonas |  |
| ASV_000051 | 0.7472 | 0.0550 | Bacteria | Proteobacteria | Gammaproteobacteria | Burkholderiales | Oxalobacteraceae | Duganella | zoogloeoides |
| ASV_000061 | 0.9507 | 0.1260 | Bacteria | Bacteroidota | Bacteroidia | Flavobacteriales | Flavobacteriaceae | Flavobacterium |  |
| ASV_000063 | 0.8767 | 0.0111 | Bacteria | Proteobacteria | Gammaproteobacteria | Pseudomonadales | Pseudomonadaceae | Pseudomonas |  |
| ASV_000086 | 0.7573 | 0.0578 | Bacteria | Proteobacteria | Gammaproteobacteria | Burkholderiales | Oxalobacteraceae | Janthinobacterium |  |
| ASV_000091 | 0.6580 | 0.0304 | Bacteria | Proteobacteria | Gammaproteobacteria | Burkholderiales | Oxalobacteraceae | Rugamonas | rubra |
| ASV_000106 | 0.5747 | 0.0241 | Bacteria | Actinobacteriota | Actinobacteria | Kineosporiales | Kineosporiaceae | Kineosporia | rhamnosa |
| ASV_000169 | 0.9054 | 0.0289 | Bacteria | Proteobacteria | Gammaproteobacteria | Burkholderiales | Oxalobacteraceae | Undibacterium |  |
| ASV_000262 | 0.6849 | 0.0107 | Bacteria | Proteobacteria | Gammaproteobacteria | Burkholderiales | Comamonadaceae | Polaromonas |  |
| ASV_000323 | 0.8209 | 0.0309 | Bacteria | Firmicutes | Clostridia | Clostridiales | Clostridiaceae | Clostridium sensu stricto 1 |  |
| ASV_000600 | 0.9500 | 0.0206 | Bacteria | Bacteroidota | Bacteroidia | Cytophagales | Microscillaceae |  |  |
| ASV_000706 | 0.7321 | 0.0107 | Bacteria | Bacteroidota | Bacteroidia | Chitinophagales | Chitinophagaceae | Niastella |  |
| OTU_2 | 1.0000 | 0.0125 | Fungi | Ascomycota | Dothideomycetes | Pleosporales | Phaeosphaeriaceae | Ophiosphaerella |  |
| OTU_3 | 0.8398 | 0.2893 | Fungi | Ascomycota | Leotiomycetes | Helotiales | Helotiales_fam_Incertae_sedis |  |  |
| OTU_6 | 0.5890 | 0.1286 | Fungi | Ascomycota | Dothideomycetes | Pleosporales | Leptosphaeriaceae | Plenodomus |  |
| OTU_9 | 1.0000 | 0.0110 | Fungi | Ascomycota | Dothideomycetes | Pleosporales | Lindgomycetaceae | Clohesyomyces |  |
| OTU_11 | 0.5505 | 0.1183 | Fungi | Ascomycota | Leotiomycetes |  |  |  |  |
| OTU_20 | 0.6667 | 0.1006 | Fungi | Ascomycota | Leotiomycetes | Helotiales | Helotiales_fam_Incertae_sedis | Tetracladium |  |
| OTU_44 | 0.6805 | 0.0577 | Fungi | Ascomycota | Leotiomycetes | Helotiales | Helotiales_fam_Incertae_sedis | Rhexocercosporidium |  |
| OTU_71 | 0.7701 | 0.0700 | Fungi | Glomeromycota | Glomeromycetes | Entrophosporales | Entrophosporaceae | Claroideoglomus | hanlinii |
| OTU_81 | 0.6457 | 0.0142 | Fungi | Ascomycota | Orbiliomycetes | Orbiliales | Orbiliaceae |  |  |
| OTU_112 | 0.7727 | 0.0153 | Fungi | Glomeromycota | Glomeromycetes | Entrophosporales | Entrophosporaceae | Claroideoglomus | hanlinii |
| OTU_120 | 0.8467 | 0.0137 | Fungi | Ascomycota | Dothideomycetes | Pleosporales |  |  |  |
| OTU_192 | 0.7981 | 0.0108 | Fungi | Ascomycota | Leotiomycetes | Helotiales | Helotiales_fam_Incertae_sedis | Rhexocercosporidium |  |

**Tab. S6 PERMANOVA outputs for abiotic and biotic predictors of microbial community composition in Poa kerguelensis (β-diversity, Aitchison distances).** Only significant effects (p < 0.05) are reported.

|  |  |  | **Bacteria** | | | **Fungi** | | |
| --- | --- | --- | --- | --- | --- | --- | --- | --- |
|  |  | **Variable** | **F** | **R²** | **p-value** | **F** | **R²** | **p-value** |
| **Bulk soil** | *Climatic variables* | Elevation | 4.3984 | 0.08687 | 0.0001 | 3.2230 | 0.07320 | 0.0002 |
|  |  | Wind exposure index | 4.2874 | 0.08467 | 0.0001 | 3.0589 | 0.06947 | 0.0002 |
|  |  | Average soil temperature | 4.3912 | 0.08672 | 0.0001 | 3.2141 | 0.07300 | 0.0002 |
|  |  | Soil humidity | 4.6980 | 0.09278 | 0.0001 | 3.1402 | 0.07132 | 0.0001 |
|  |  | Number of freeze-thaw cycles | 4.7737 | 0.09428 | 0.0001 | 3.1687 | 0.07196 | 0.0003 |
|  | *Soil edaphic variables* | Total nitrogen | 1.7368 | 0.03960 | 0.0213 | - | - | - |
|  |  | Total carbon | 1.5884 | 0.03622 | 0.0430 | - | - | - |
|  |  | Available phosphorus (Olsen) | 2.5646 | 0.05847 | 0.0002 | 2.5756 | 0.06122 | 0.0004 |
|  |  | Ammonium | 2.1207 | 0.04835 | 0.0019 | 1.6866 | 0.04009 | 0.0153 |
|  |  | Soluble sulfur | 2.5045 | 0.05710 | 0.0009 | 2.3798 | 0.05657 | 0.0006 |
|  |  | pH | 2.5132 | 0.05730 | 0.0003 | 1.8440 | 0.04383 | 0.0068 |
|  | *Morphological variables* | Largest diameter | 1.7020 | 0.04885 | 0.0391 | 1.6763 | 0.05128 | 0.0193 |
|  |  | Number of leaves | 2.0383 | 0.05850 | 0.0133 | - | - | - |
|  |  | Number of ears | 1.7723 | 0.05086 | 0.0252 | 1.5258 | 0.04667 | 0.0413 |
|  |  | Dry shoot mass | 1.6876 | 0.04843 | 0.0400 | 1.5815 | 0.04838 | 0.0273 |
| **Rhizospheric soil** | *Climatic variables* | Elevation | 4.0011 | 0.07630 | 0.0001 | 3.1208 | 0.06662 | 0.0007 |
|  |  | Wind exposure index | 3.9985 | 0.07625 | 0.0001 | 3.0614 | 0.06535 | 0.0010 |
|  |  | Average soil temperature | 3.9603 | 0.07552 | 0.0001 | 3.0055 | 0.06416 | 0.0009 |
|  |  | Soil humidity | 4.5554 | 0.08687 | 0.0001 | 3.4721 | 0.07412 | 0.0003 |
|  |  | Number of freeze-thaw cycles | 4.8564 | 0.09261 | 0.0001 | 3.6538 | 0.07800 | 0.0002 |
|  | *Soil edaphic variables* | Total nitrogen | 1.8064 | 0.04063 | 0.0178 | 1.6055 | 0.03710 | 0.0360 |
|  |  | Total carbon | 1.6627 | 0.03740 | 0.0338 | - | - | - |
|  |  | Available phosphorus (Olsen) | 2.7788 | 0.06250 | 0.0004 | 3.1779 | 0.07343 | 0.0002 |
|  |  | Ammonium | 2.6436 | 0.04593 | 0.0043 | 1.6352 | 0.03778 | 0.0286 |
|  |  | Soluble sulfur | 2.8204 | 0.06344 | 0.0005 | 3.2144 | 0.07427 | 0.0001 |
|  |  | pH | 2.1811 | 0.04906 | 0.0028 | 1.8350 | 0.04240 | 0.0109 |
|  | *Morphological variables* | Height | - | - | - | 1.4607 | 0.04493 | 0.0467 |
|  |  | Largest diameter | 1.6818 | 0.04995 | 0.0353 | 1.8689 | 0.05748 | 0.0058 |
|  |  | Number of leaves | 1.6459 | 0.04889 | 0.0391 | 1.6147 | 0.04966 | 0.0218 |
|  |  | Dry shoot mass | 1.7280 | 0.05133 | 0.0317 | 1.7223 | 0.05297 | 0.0113 |
| **Root** | *Climatic variables* | Elevation | 1.4362 | 0.04639 | 0.0083 | 2.0127 | 0.05786 | 0.0008 |
|  |  | Wind exposure index | 1.4153 | 0.04572 | 0.0126 | 2.0046 | 0.05763 | 0.0007 |
|  |  | Average soil temperature | 1.4464 | 0.04672 | 0.0073 | 2.0166 | 0.05797 | 0.0007 |
|  |  | Soil humidity | 1.3698 | 0.04425 | 0.0276 | 1.7423 | 0.05009 | 0.0050 |
|  |  | Number of freeze-thaw cycles | 1.3489 | 0.04357 | 0.0232 | 1.6648 | 0.04786 | 0.0068 |
|  | *Soil edaphic variables* | Available phosphorus (Olsen) | - | - | - | 1.9682 | 0.05537 | 0.0006 |
|  |  | Soluble sulfur | 1.5335 | 0.06432 | 0.0006 | 2.3250 | 0.04490 | 0.0055 |
|  |  | pH | - | - | - | 1.0662 | 0.03615 | 0.0396 |
|  | *Morphological variables* | Largest diameter | - | - | - | 1.6618 | 0.04954 | 0.0238 |
|  |  | Number of leaves | 1.4634 | 0.04477 | 0.0365 | 1.5281 | 0.04555 | 0.0401 |
|  |  | Dry shoot mass | 1.4486 | 0.04432 | 0.0339 | 2.0557 | 0.06128 | 0.0033 |
|  |  | Dry root mass | 1.6334 | 0.04997 | 0.0432 | - | - | - |
| **Leaf** | *Climatic variables* | Elevation | 2.3876 | 0.05385 | 0.0063 | - | - | - |
|  |  | Wind exposure index | 5.7099 | 0.12879 | 0.0001 | - | - | - |
|  | *Soil edaphic variables* | Soluble sulfur | 1.5505 | 0.05742 | 0.0041 | - | - | - |
|  |  | pH | 1.8011 | 0.06670 | 0.0022 | - | - | - |
|  | *Morphological variables* | Largest diameter | 2.0509 | 0.06135 | 0.0212 | - | - | - |
|  |  | Dry shoot mass | 2.4344 | 0.07282 | 0.0068 | - | - | - |
| **Leaf Surface** | *Climatic variables* | Elevation | 4.3560 | 0.12580 | 0.0001 | - | - | - |
|  |  | Wind exposure index | 3.3693 | 0.09730 | 0.0001 | - | - | - |
|  |  | Average soil temperature | 4.3486 | 0.12558 | 0.0001 | - | - | - |
|  | *Soil edaphic variables* | Available phosphorus (Olsen) | 2.1326 | 0.07867 | 0.0025 | - | - | - |
|  |  | Ammonium | 1.6570 | 0.06113 | 0.0349 | - | - | - |
|  |  | Soluble sulfur | 1.6210 | 0.05980 | 0.0192 | - | - | - |
|  | *Morphological variables* | Dry shoot mass | 1.4764 | 0.04607 | 0.0422 | - | - | - |

**Tab. S7 PERMANOVA outputs for abiotic and biotic predictors of microbial composition in *Pringlea antiscorbutica* (β-diversity, Aitchison distances).** Only significant effects (p < 0.05) are reported.

|  |  |  | **Bacteria** | | | **Fungi** | | |
| --- | --- | --- | --- | --- | --- | --- | --- | --- |
|  |  | **Variable** | **F** | **R²** | **p-value** | **F** | **R²** | **p-value** |
| **Bulk soil** | *Climatic variables* | Elevation | 4.1165 | 0.17418 | 0.0001 | 2.5852 | 0.11844 | 0.0001 |
|  |  | Wind exposure index | 4.2482 | 0.18642 | 0.0001 | 2.8658 | 0.13129 | 0.0001 |
|  |  | Average soil temperature | 4.5316 | 0.18218 | 0.0001 | 2.4258 | 0.11113 | 0.0002 |
|  | *Morphological variables* | Number of leaves | 2.1773 | 0.11253 | 0.0048 | 1.6895 | 0.09082 | 0.0134 |
|  |  | Diameter at collar | 1.9753 | 0.10209 | 0.0089 | 1.8950 | 0.10186 | 0.0039 |
|  |  | Maximum root diameter | - | - | - | 1.7929 | 0.09638 | 0.0061 |
| **Rhizospheric soil** | *Climatic variables* | Elevation | 4.1337 | 0.17126 | 0.0001 | 2.8656 | 0.13083 | 0.0001 |
|  |  | Wind exposure index | 4.1522 | 0.17203 | 0.0001 | 3.0177 | 0.13778 | 0.0001 |
|  |  | Average soil temperature | 3.5543 | 0.14626 | 0.0001 | 2.4715 | 0.11284 | 0.0002 |
|  | *Morphological variables* | Diameter at collar | - | - | - | 1.4234 | 0.07211 | 0.0490 |
| **Root** | *Climatic variables* | Elevation | - | - | - | 1.5658 | 0.07782 | 0.0250 |
|  |  | Wind exposure index | 1.4989 | 0.07687 | 0.0176 | 1.6561 | 0.08231 | 0.0096 |
|  | *Soil edaphic variables* | Soluble sulfur | - | - | - | 2.3250 | 0.13431 | 0.0225 |
|  | *Morphological variables* | Largest diameter | 1.2862 | 0.06483 | 0.0291 | - | - | - |
|  |  | Number of leaves | 1.4612 | 0.07365 | 0.0042 | - | - | - |
| **Leaf surface** | *Climatic variables* | Elevation | 1.5091 | 0.07029 | 0.0024 | - | - | - |
|  |  | Wind exposure index | 1.5389 | 0.07128 | 0.0050 | - | - | - |
|  |  | Average soil temperature | 2.0192 | 0.09405 | 0.0001 | - | - | - |

**References**

1. Frenot Y. Interactions entre la faune lombricienne et les systèmes édaphiques d’une île subantarctique : île de la possession, Archipel Crozet. Université de Rennes 1; 1986.

2. Frenot Y, Vliet-Lanoe BV, Gloaguen J-C. Particle Translocation and Initial Soil Development on a Glacier Foreland, Kerguelen Islands, Subantarctic. Arctic and Alpine Research. 1995;27:107.

3. Cramer MD, Hedding DW, Greve M, Guy MF, Ripley BS. Plant specialisation may limit climate-induced vegetation change to within topographic and edaphic niches on a sub-Antarctic island. Functional Ecology. 2022;36:2636–48.
